# Unveiling the Mechanism of Arginine Transport through AdiC with Molecular Dynamics Simulations: The Guiding Role of Aromatic Residues

**DOI:** 10.1371/journal.pone.0160219

**Published:** 2016-08-02

**Authors:** Eva-Maria Krammer, Kassem Ghaddar, Bruno André, Martine Prévost

**Affiliations:** 1 Structure et Fonction des Membranes Biologiques, Université Libre de Bruxelles (ULB), Brussels, Belgium; 2 Molecular Physiology of the Cell, Université Libre de Bruxelles (ULB), IBMM, Gosselies, Belgium; University of Bern, SWITZERLAND

## Abstract

Commensal and pathogenic enteric bacteria have developed several systems to adapt to proton leakage into the cytoplasm resulting from extreme acidic conditions. One such system involves arginine uptake followed by export of the decarboxylated product agmatine, carried out by the arginine/agmatine antiporter (AdiC), which thus works as a virtual proton pump. Here, using classical and targeted molecular dynamics, we investigated at the atomic level the mechanism of arginine transport through AdiC of *E*. *coli*. Overall, our MD simulation data clearly demonstrate that global rearrangements of several transmembrane segments are necessary but not sufficient for achieving transitions between structural states along the arginine translocation pathway. In particular, local structural changes, namely rotameric conversions of two aromatic residues, are needed to regulate access to both the outward- and inward-facing states. Our simulations have also enabled identification of a few residues, overwhelmingly aromatic, which are essential to guiding arginine in the course of its translocation. Most of them belong to gating elements whose coordinated motions contribute to the alternating access mechanism. Their conservation in all known *E*. *coli* acid resistance antiporters suggests that the transport mechanisms of these systems share common features. Last but not least, knowledge of the functional properties of AdiC can advance our understanding of the members of the amino acid-carbocation-polyamine superfamily, notably in eukaryotic cells.

## Introduction

Commensal and pathogenic enteric bacteria must adapt to an extreme acidic environment as they transit through the stomach to colonize the intestine. To withstand the deleterious effects of acid stress, they often use a combination of protective molecular mechanisms, one of which relies on activation of very potent proton-consuming systems that help maintain a steady-state intracellular pH [1–3]. The basic principle of these acid resistance (AR) systems consists in the uptake of specific amino acids and, in most cases, in their decarboxylation by cytosolic enzymes. These consume one intracellular proton per decarboxylation reaction, which is followed by export of the decarboxylated product. Two major protein components, an inner-membrane substrate/product antiporter functioning as a virtual proton pump and a cytosolic decarboxylase, act together to perform these tasks. In *E*. *coli*, four such AR systems have been characterized: the ornithine/putrescine, lysine/cadaverine, arginine/agmatine (Agm), and glutamate/γ-aminobutyric acid (GABA) systems [4–7]. The first two work under moderate pH stress, whereas the last two operate in an extreme acidic environment. An additional *E*. *coli* AR system has recently been discovered, which imports glutamine into the cytosol, where it is converted to glutamate by an acid-activated ammonia-releasing amidohydrolase [8]. The resulting ammonia then combines at low pH with intracellular protons, raising thereby the intracellular pH, while glutamate, depending on the acidity of the medium, might be converted to GABA and subsequently exported by its antiporter [8,9].

The four inner-membrane *E*. *coli* AR antiporters all belong to the amino-acid-polyamine-organocation (APC) superfamily, the second largest superfamily of secondary transporters [10]. This superfamily is characterized by a fairly large number of atomic level structures of proteins, belonging to different families, which, despite sharing low sequence identity, adopt a common fold, named ‘LeuT fold’ after the first crystallized protein found to adopt this fold [11–13]. The LeuT fold consists of a core of two inverted repeats of five helical transmembrane (TM) segments that intertwine to form an α-helical subdomain. Additional helical TM segments occur at either the N- or the C-terminus. The first repeat is related to the second one by a pseudo twofold symmetry around an axis through the center of the membrane plane. The middle of the core formed by these two inverted repeats, approximately halfway across the membrane bilayer, is the location of a binding site for substrates [14–24]. The APC structures notably include those of two AR antiporters, the arginine/Agm (AdiC) [14,15,25,26] and glutamate/GABA (GadC) [27] transporters.

3D structures of APC members have been trapped in different conformational states. They reveal that transport occurs, as in other secondary transporters, via alternating access to the substrate binding site, caused by conformational changes to either side of the membrane [28]. They also demonstrate that the 5-TM inverted-topology repeats play a central role in this alternating-access transport mechanism [12,29]. According to this model, the substrate (and the co-substrate ion for symporters) binds to an outward-facing (OF) conformation of the transporter. Following substrate binding, access to the binding site is closed on the OF side, and this closure promotes a transition to an inward-facing (IF) state. The substrate is then released and the transporter returns to its original conformation. In ion-coupled symporters this transition occurs with a substrate-free binding site, while in antiporters, another substrate binds and is transported in the opposite direction. The alternating access model thus entails the occurrence of at least two major conformational states of the protein, the OF state, which is accessible on the outer side of the membrane, and the IF state, which is accessible on the opposite side of the membrane. Additional intermediate states may exist along the conformational transition from OF to IF or vice versa, as suggested by several crystal structures determined for APC transporters [14–18,20,21,23,25,26,30–38], though it is unclear how populated these potential states are.

The transitions occurring during transport are often depicted in terms of opening and closure of ‘gates’ which completely or partially occlude the binding site. These gates can consist of portions of TM segments and/or of a few amino acids [13,29,39]. For AR transporters it has been proposed, mainly on the basis of AdiC crystal structures, that substrate transport could be controlled by three gates: a proximal gate involved in closure on the OF side, a distal gate, and a middle gate whose opening would induce opening of the distal gate and switching of the transporter to an IF conformation [14].

So far a complete picture of the transport mechanism of APC transporters remains elusive. BetP [24,35,37,38], LeuT [40], and Mhp1 [18,22,31] are the APC members whose transport mechanisms are most comprehensively characterized structurally. A paucity of structural snapshots and the difficulty of characterizing different intermediate structures may explain the less detailed pictures available for other APC transporters. For these transporters, we thus lack details of structural responses, for instance upon substrate binding/release, and of the transitions leading from the OF to the IF state. Molecular dynamics (MD) simulation techniques are a powerful means of understanding the mechanics of all relevant steps of substrate binding and release and of the full substrate translocation pathway with its conformational transitions [41]. Large-scale protein motions needed for such conformational transitions are in most cases beyond the scope of classical MD simulations and can only be assessed by non-equilibrium MD methods. Though targeted MD (tMD) and steered MD methods were recently challenged in one study of an ABC transporter [42] they are still the most widely used non-equilibrium methods which provide a mechanistic picture for conformational transitions in transporters without compromising the atomic representation [41]. For instance the conformational transitions of a few LeuT-fold transporters were investigated using steered MD, tMD, accelerated MD and metadynamics [43–49].

As regards the AR antiporters, the AdiC structures trapped in the arginine-free or arginine-bound OF open conformations and those trapped in the occluded arginine-bound conformation [14,15,25,26], along with the structure of GadC determined in an IF open substrate-free conformation [27], open the way to using MD to study the mechanism of substrate transport in AR systems at the atomic level. To the best of our knowledge, so far only a few MD and modeling studies have been performed on AdiC [15,50,51]. Notably, an MD study exploiting crystallographic and docking data has pointed to the involvement of several residues in proper coordination of the ligand and in occlusion of the binding site to the OF open state [15]. However, a complete AR transporter transport cycle has yet to be investigated. In the present study, we have simulated arginine transport from the outside medium to the cytosol, using a combination of targeted MD (tMD) and classical MD simulations. We have modeled translocation as successive steps simulating the sequential conformational changes undergone by the transporter. These transitions were guided along the translocation path through conformations depicted by different crystal structures. Overall, our MD simulation data clearly demonstrate that concomitant rearrangements of TM1 and TM6 backbone segments are necessary but insufficient to promote the transition from the OF to the IF state. They stress the fundamental role of local conformational changes, in particular that of rotamer transitions in two aromatic gating residues, in spurring the structural changes that regulate accessibility of the substrate binding site. Our study also highlights that a few additional residues, mostly aromatic residues and one acidic one, are essential to assisting the translocation of arginine. The mechanism uncovered in this study suggests that all AR transporters, and possibly other members of the APC superfamily, might share a common transport mechanism, although some features are likely to differ.

## Materials and Methods

### Molecular dynamics simulation details and protocols

All MD simulations were performed in the isothermal-isobaric ensembles at 300 K with the program NAMD2.9 [52]. The CHARMM27 force field [53,54] with CMAP corrections [55] was used to describe protein, water and ion atoms. A united atom force field described the lipids [56]. Details of the setups for all molecular systems are given in S1 File. The protocols of the conventional MD simulations of the AdiC and GadC crystal structures are described in S1 File.

### Simulations of arginine transport starting from the AdiC OF open state

**Fig 1:**
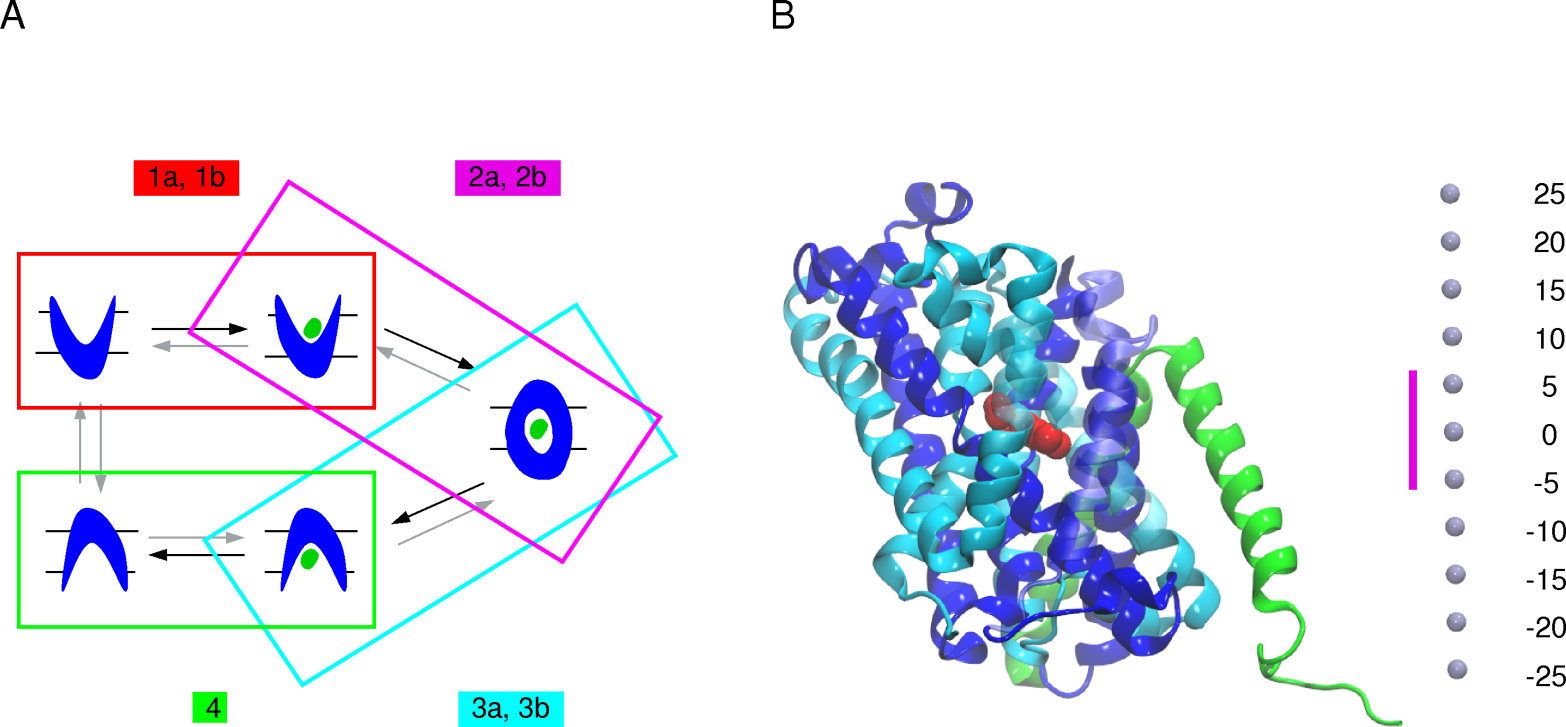
Schematic representation of arginine transport by the AdiC antiporter. (A) The reconstruction of the arginine transport based on tMDs followed by relaxation MD simulations and starting from the AdiC OF open state is shown. (Step 1) Arg^+^ binding to the OF open state is simulated by means of either conventional MD simulations (step 1a) or tMDs targeting the location of arginine in the OF open substrate-bound crystal structure (PDB ID: 3OB6 [15]) (step 1b). (Step 2) Occlusion of the OF state is modeled by targeting the occluded substrate-bound crystal structure (PDB ID: 3L1L [14]) including (step 2a) or not (step 2b) the Trp202 side chain among the targeted protein portions. (Step 3) The transition from the occluded substrate-bound to the IF open substrate-bound state is simulated, targeting either one (step 3a) or each of the two (step 3b) IF crystal structures of GadC (PDB IDs: 4DJI and 4DJK [27]) including (step 3a) or not (step 3b) the Trp293 side chain in the targeted protein portions (for further details Material and Methods and S1 Table). (Step 4) Release of the arginine substrate starting from the final conformation of the tMDs obtained from step 3b. Black arrows indicate the migration direction of the arginine substrate. (B) AdiC structure. The protein structure (PDB ID: 3OB6) is depicted as a cartoon. The two inverted repeats made of 5 helical TM segments shaping the LeuT fold are colored, one in blue and the other in cyan. TM11 and TM12 (in green) of each monomer form the dimerization interface. The bound substrate is shown as red van der Waals spheres. The TM segments overlying the binding site are shown as transparent cartoon. Light blue dots shown on the right side represent the scale along the transporter main axis that is perpendicular to the membrane. The location of the binding site, highlighted by a magenta bar, is defined as the region encompassed between Trp202 and Trp293 Cα atoms.

The transport of arginine was simulated as several successive steps summarized in Fig 1A and S1 Table. To investigate, in the first step, the binding of Arg^+^ to the OF open unbound state, we used two strategies. The first consisted in performing 10-ns conventional simulations with Arg^+^ initially placed on the top of the transporter (Fig 1A: step 1a). The second relied on 5-ns tMD simulations during which the arginine substrate was guided down to the binding site using the position of Arg in the OF open arginine-bound structure (PDB ID: 3OB6 [15]) as a target and applying a weight of 1.0 kcal/mol/Å^2^ on each heavy atom of the substrate (Fig 1A: step 1b). The orientation of the arginine side chain differs in the two monomers of the 3OB6 structure. We chose monomer A as the target, since the position of its arginine is the most similar to that in the occluded structure (PDB ID: 3L1L [14]), though slightly shifted up towards the OF side. Two different protein conformations extracted from a 10-ns conventional MD of the OF open substrate-free crystal structure (see section "Simulations of AdiC and GadC crystal structures" in S1 File) and three initial positions of arginine substrate at the mouth of the OF open funnel were used, resulting in a total of 12 binding trajectories achieved in each monomer of the simulated dimers. Every tMD was followed by a 5-ns conventional simulation to relax the system.

To model the occlusion of the OF open substrate-bound state (Fig 1A: step 2), 10-ns tMD simulations were performed starting from the end conformation of the relaxation simulations of step 1b, using as target the crystal structure of the occluded bound state (PDB ID: 3L1L). A weight of 0.5 kcal/mol/Å^2^ was applied to the backbone heavy atoms of TM1 (residues 22 to 38), TM2 (residues 41 to 59), TM6 (residues 195 to 205), portions of the loops between TM1 and TM2 (residues 39 and 40), and between TM9 and TM10 (residues 325 to 360) (Fig 1A: step 2a). These protein portions were selected after examining a superimposition of the OF open structure onto the occluded structures of AdiC (PDB IDs: 3OB6, 3LRB, 3NCY versus 3L1L [14,15,25,26]) and of LeuT (3TT1 versus 2A65 [16,30]). tMD simulations were also performed adding the Trp202 side chain to the ensemble of targeted atoms defined in the previous tMDs (Fig 1A: step 2b). A weight of 1.5 kcal/mol/Å^2^ was applied to the Trp202 side chain heavy atoms. A 5-ns conventional MD simulation followed each tMD (Fig 1A: step 2a or 2b).

The transition from the occluded to the IF open substrate-bound state was simulated during 15-ns tMD simulations starting from the final conformation of the relaxation simulations of step 2b (Fig 1A: step 3a or 3b). As at the time of the study no 3D structure of AdiC had been determined in an IF state we examined all crystal structures of APC-superfamily amino acid transporters determined in an IF open state (PBD IDs: 3TT3, 4DJI, 4DJK) [23,27] and compared them to different occluded structures of amino acid transporters in this superfamily, namely those of AdiC, LeuT, and MhsT (PDB IDs: 3L1L, 2A65 and 4US3) [14,16,23], to evidence the structural elements undergoing a significant change between these states. By superimposing monomer A of the two AR GadC structures trapped in an IF state (PDB IDs: 4DJI/4DJK) [27] onto the occluded conformation of AdiC (Fig 1A: step 2b), we then defined the protein portions to be targeted in step 3. To reach the IF open state, a weight of 0.5 kcal/mol/Å^2^ was thus applied to the backbone heavy atoms of TM1 (residues 12 to 24), TM2 (residues 39 to 64), TM6 (residues 204 to 210), and TM7 (residues 222 to 250) (Fig 1A: step 3a) of AdiC, and the positions of the corresponding residues in monomer A of one of the two GadC structures (PDB ID: 4DJI) [27] were targeted. Three different tMD simulations were performed (S1 Table) and each was followed by a 5-ns conventional MD relaxation simulation. tMD simulations were also performed, with the side chain of Trp293, corresponding to Trp308 in GadC, added to the ensemble of targeted atoms (Fig 1A: step 3b). As the rotameric state of Trp308 differs in the two different GadC crystal structures, both were used as templates and a weight of 1.5 kcal/mol/Å^2^ was applied to the Trp293 side-chain heavy atoms. Targeting of two different GadC structures in this case led to a total of 24 simulated transitions from the occluded to the IF open state in 12 dimers (S1 Table). Each tMD simulation was followed by a 5-ns conventional relaxation simulation.

To model the release of the arginine, 10-ns tMD simulations were performed, starting from the final conformations of the tMDs obtained in step 3b and targeting Arg^+^ randomly positioned in three different manners in the intracellular medium (Fig 1A: step 4). A weight of 1 kcal/mol/Å^2^ was applied to all heavy atoms of the ligand. Targeting the three different Arg^+^ positions led to a total of 72 simulated OF-to-IF transitions. Each tMD simulation was followed by a 5-ns conventional relaxation simulation.

### Simulations of truncated arginine transport starting from the AdiC occluded state

**Fig 2:**
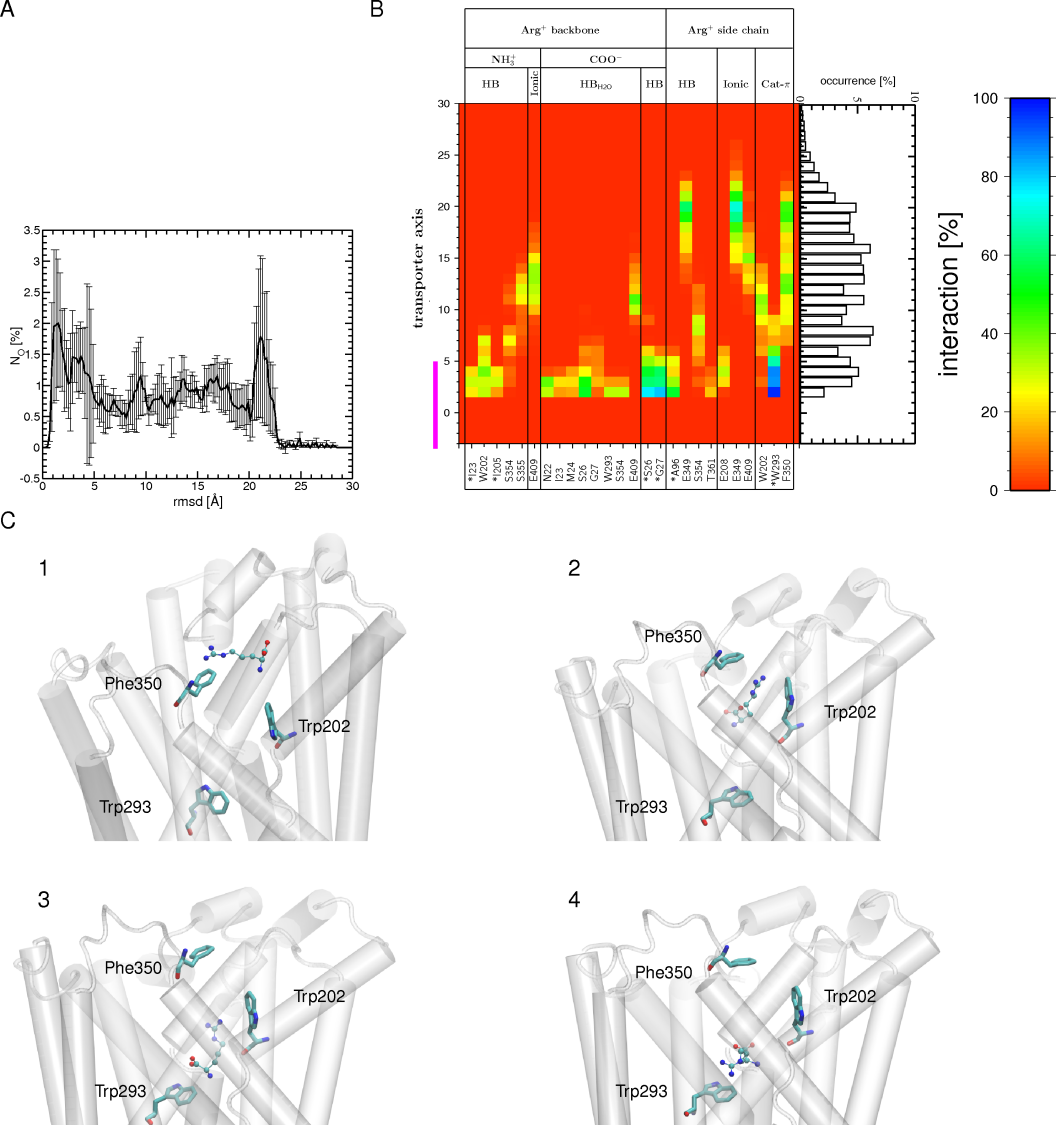
Binding of Arg^+^ to the OF open state during the tMDs. (A) Number of occurrences (N_O_) for finding the substrate at a certain RSMD value, computed for the carbon atoms of Arg^+^ using all 6 tMD trajectories (each containing two AdiC monomers) and its crystal position in the OF open crystal structure (PDB ID 3OB6; Monomer A) as a reference. An RMSD value of zero Å corresponds to a perfect match between the MD conformation and the targeted crystal position of arginine. The standard error is shown as black bars. (B) Interactions (H bonds, ionic and cation-π interactions) formed between the Arg^+^ backbone or side chain and protein residues during its migration down to the binding site are shown, along with the occurrence of observing the center of mass of Arg^+^ at a certain position along the main axis of the transporter between the external medium and the binding site, as depicted by a bar graph representation. Only interactions with an occurrence higher than 20% in at least one bin width from all 12 binding events of the 6 tMD trajectories are shown. The abbreviations used for the different interactions are: ionic for ionic interaction, HB for H bond, HB_H2O_ for water-mediated H bond, and Cat-π for cation π interaction. The binding site region (-5 to 5 Å) is highlighted by a magenta bar. (C) Four tMD conformations of AdiC highlighting Arg^+^ migration (1–4). The Arg^+^ side chain first forms a cation-π interaction with Phe350 (1). It then moves further down to form a sandwich configuration between the Phe350 and Trp202 side chains (2). Following this configuration, Arg^+^ slides down towards the binding site, forming a cation-π interaction with Trp202 (3). Arg^+^ leaves Trp202 to form a cation-π interaction with Trp293, which shapes the bottom of the binding site. Residues making persistent interactions with Arg^+^ are shown as sticks and the protein portion shaping the OF side is shown as a white cartoon. The Arg^+^ substrate is depicted as CPK.

Translocation of arginine was also performed (S1A Fig step 5 and 6; S2 Table) starting from the end conformation of an MD simulation performed on the occluded crystal structure of AdiC (see S1 File) following the same protocol as described above for steps 3b and 4 (Fig 2A).

### Analysis of the MD trajectories and crystal structures

The quality of the classical MD simulations was assessed on the basis of the time-dependent root mean square deviations (RMSDs) and fluctuations (RMSFs) of the backbone heavy atom positions computed with VMD [57]. Prior to the RMSF calculation the protein in each frame of the trajectories was aligned onto the first protein conformation of the trajectory. As for the RMSD calculations all conformations of the protein were aligned to the crystal structure used as a reference. Missing residues in the crystal structures were not included in the RMSD calculation. Monomer A of the OF open crystal structure (PDB ID: 3OB6) and of the IF GadC (PDB ID: 4DJI) were used as reference structures. For the tMD simulating the transition from occluded to IF open (step 3b in Fig 1A) the time evolution of the restraint RMSD potential was computed.

To monitor the OF-to-IF conformational transitions, the occurrence of open channels was searched using the HOLE program [58]. This program computes the radius profile of a pore by determining the largest possible radius of a sphere whose center is moved in a random fashion at different particular planes along the a vector describing the pore channel direction [58]. In the present study the transporter funnel radius was computed over the range -15 to 15 Å along its inertia main axis aligned with the z axis normal to the membrane (Fig 1B). The location of the AdiC binding site was defined as being between the Cα atoms of Trp202 and Trp293 and spans roughly the region of -5 to 5 Å along the transporter main axis.

The radius was averaged over 100 configurations extracted from the last 0.1 ns of the MD trajectories. The transporter funnel radius was also calculated for the crystal structures of different amino acid transporters of the APC superfamily (PDB IDs: 3OB6, 3L1L, 4DIJ, 3GI8, 2A65, 3TT3, 3TT1, 4US3; S2A Fig) [9,14–16,23,30,33]. For the dimeric structures, the first chain was used.

Several types of interactions between the arginine substrate and the protein residues were monitored in all trajectories and crystal structures, using VMD [57] (for ionic interactions) and EUCB [59] (for cation-π interactions and direct and water-mediated hydrogen bonds (H bonds). The different types of interactions and their parameters are listed in S5 Table. The probability of an interaction being formed by the arginine substrate was estimated along the main axis of the transporter in 1-Å-thick slices. The interaction probability was calculated as the number of snapshots featuring a given interaction and having the center of mass of Arg^+^ located in a given slice divided by the total number of snapshots having the center of mass of Arg^+^ located in the same slice. In the interactions plots the interactions formed with a probability higher or equal to 20% over all MD trajectories in at least one thickness slice along the main axis were shown.

VMD [53] was used to calculate the RMSD of the arginine substrate position, using as reference its position in either the OF open or the occluded crystal structure and its carbon atoms. Prior to the RMSD calculation the protein in each frame of the trajectories was translated to its center of mass. For the AdiC OF open crystal structure, featuring in the dimer two different positions of the arginine side chain, we used the structure with the smaller RMSD with respect to the position of arginine in the occluded crystal structure (monomer A). The standard error was calculated by averaging the total trajectory over several bins (3 for steps 1b and 2b, 6 for step 3b, 18 for step 4, and 3 for step 5). All images depicting the protein were prepared using VMD [57].

## Results

### Characterization of the states adopted by the crystal structures

To simulate arginine transport through AdiC, we first characterized the conformational states of the crystal structures of APC-superfamily amino acid transporters by computing the corresponding funnel radius profiles (S2 Fig). The OF and IF open crystal structures show a profile with a wide opening, on the OF or IF side, respectively, and an occlusion on the opposite side, in keeping with the assigned state (S2B and S2C Fig). The situation is quite different for the structures reported as OF or IF open occluded states the funnel radius profiles indicate that several of these structures, including the AdiC structure, are closed on both sides (S2D Fig). Such a structure constitutes an almost fully symmetrical occluded arrangement. As this AdiC structure is that of a double mutant [25], we substituted the two wild-type residues for the two mutated ones. A 10-ns MD simulation (S1B Fig: simulation C) of this structure featured a low backbone RSMD of 1.5 Å (S3A Fig) and a fairly good correlation of its RMSF with the secondary structure content (S3B Fig). The funnel radius profile computed from the last portion of this simulation depicts, as for the crystal structure (S2C Fig), a state with the OF and IF vestibules collapsed on both sides (S3C Fig), corresponding to a quasi-symmetrically occluded intermediate between an OF and an IF state along the arginine transport sequence. Though the use of more local properties could lead to slightly different assignments, we adopted the funnel radius as the feature describing the structural state of the transporter structures. In the light of the data just described, we simulated the transport of arginine through AdiC on the basis of a transport sequence featuring a single occluded intermediate between the OF and IF states (Fig 1A).

### Simulated arginine transport

AdiC is reported mainly to mediate Arg^+^/Agm^2+^ exchange to support effective virtual proton pumping [60]. Using MD simulations, we thus investigated the molecular mechanism of the complete transport of the Arg^+^ by AdiC. Arginine translocation was studied by breaking down the whole process into several consecutive steps (Fig 1A). Step 1, i.e. arginine migration along the OF open funnel towards the binding site, was simulated using the arginine-bound OF open structure as a target conformation. Step 2, i.e. closure of the binding site, was modeled starting from the last conformation of the arginine-bound OF open state simulations performed in step 1 and using the occluded arginine-bound structure as a target conformation. Step 3, i.e. the transition from the occluded state to the IF state, was simulated using as guide the IF open structure of the GadC AR antiporter, as no structure of AdiC in an IF state has been determined so far. Step 4, i.e. arginine release from the IF state in the cytosol, simulates the last step of the transport. The complete translocation of arginine was monitored along the multiple trajectories of the different simulated steps in terms of structural properties of the substrate and transporter and of interactions between the substrate and transporter residues.

#### Binding of arginine to the OF open state

Before simulating arginine binding to the AdiC OF open crystal structure, we checked, in a 10-ns MD simulation, the stability of this structure after removal of the arginine substrate (S1B Fig: simulation A). In these simulations, the time-averaged backbone RMSD is fairly low (about 1.3 Å; S3D Fig) and the backbone positional RMSF (S3E Fig) correlate well with the secondary structure content. The profile of the funnel opening (S3F Fig) shows that the transporter remains in an OF open state during the simulation, despite the removal of the arginine substrate. All these observations suggest that the OF open substrate-unbound structure is stable throughout the MD simulation.

This being established, we then explored, in six different conventional MD simulations, the pathway followed by Arg^+^ during its migration from the outside medium to the binding site (Fig 1A: step 1a). Quite expectedly, none of these trajectories showed migration of Arg^+^ through the OF funnel to its binding site. Thus, to address the issue of arginine binding, we performed MD simulations in which the Arg^+^ substrate was guided to its binding pocket, using as target the position of arginine in the OF open arginine-bound crystal structure (Fig 1A: step 1b) and starting as in the conventional MDs from six different initial molecular systems. In all 12 monomers of the six tMD simulations, the arginine substrate moves downhill towards its binding site, as shown by its RMSD (Fig 2A). All end conformations but one feature the Arg^+^ substrate positioned similarly in the binding pocket.

**Fig 3:**
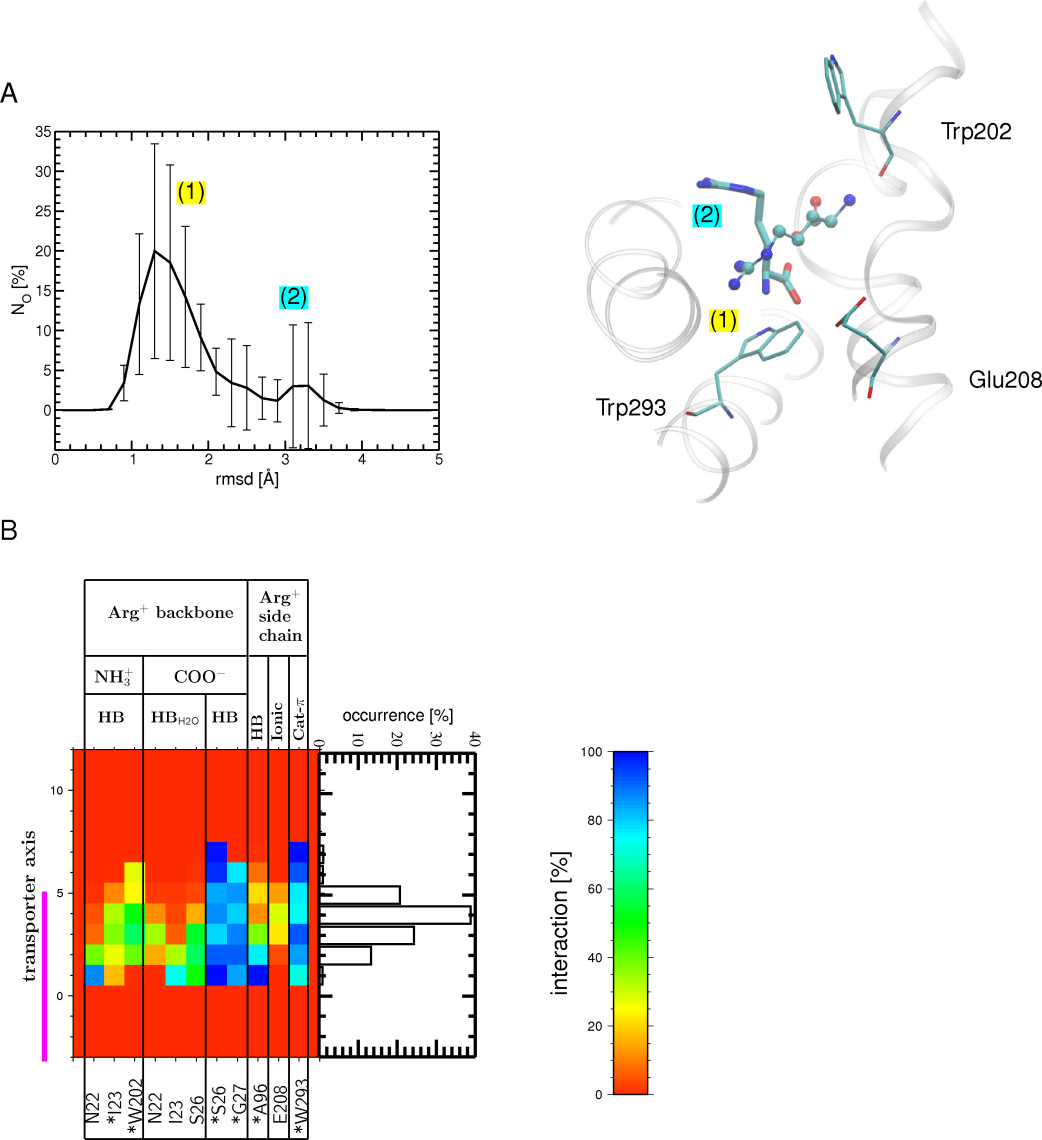
Arg^+^ bound to the OF open state after the binding stage and during the relaxation MDs. (A) Number of occurrences (N_O_) for finding the substrate at a certain RSMD value, computed for the carbon atoms of Arg^+^ using all MD relaxation simulations and its crystal position in the OF open state (PDB ID: 3OB6) as a reference (left). The standard error is shown as bars. A global and a local maxima are found (labeled (1) and (2)), which describe the most populated Arg^+^ positions as depicted on the right: the major peak corresponds (1) to a binding position shown as balls-and-sticks, similar to that in the crystal structure (PDB ID 3OB6, monomer A); the minor peak (2), shown as sticks, indicates a different position, in which the substrate is rotated relative to position (1). Regions surrounding the binding site (white ribbons) as well as a few binding site residues (atom type colored sticks) are also shown. (B) Interactions (H bonds, ionic and cation-π) formed during the relaxation MD simulations between the Arg^+^ backbone or side chain and protein residues in the binding site (the abbreviations used for the different interactions are listed in the legend of Fig 3). Only interactions with an occurrence higher than 20% in at least one bin width from all 12 binding events of the 6 tMD trajectories are shown. The binding site region (-5 to 5 Å) is highlighted by a magenta bar. The interactions formed in the OF open substrate-bound crystal structure (PDB ID: 3OB6 [15]) are indicated by asterisks.

In the very early stages of the downhill move, no common pathway for Arg^+^ is observed in the 12 monomers (Fig 2B; S4A Fig). Further down along the OF funnel, preferred interactions made by the arginine side chain are identified, in particular with Glu349, Phe350, and Glu409 (Fig 2B). Glu349 and Phe350 pertain to the loop modeled in one of the two monomers using that of the other monomer as template (see Material and Methods). In the latter, Phe350 points away from the transporter funnel, whereas Glu349 faces it. Remarkably, Phe350 reorients itself towards the funnel in both monomers during the 10-ns conventional MD simulation of the OF open structure of AdiC. It remains in this orientation throughout these tMDs, allowing a cation-π interaction with the migrating Arg^+^. Notably, the position reached by Phe350 is close to that observed in the OF open substrate-free crystal structure (PDB ID: 3LRB [25]) (S4B Fig). Arg^+^ subsequently enters the binding site, with its side chain first making a transient cation-π interaction with Trp202 in all simulations (Fig 2B and 2C). Its positioning in the binding pocket is guided by H bonds formed by its side chain, sequentially with the Ser354 side chain and the Ala96 backbone (Fig 2B). In the binding site, the Arg^+^ forms most of the interactions observed in the targeted OF open crystal structure (Fig 3C).

To assess the stability of Arg^+^ in its binding site, we performed 5-ns relaxation MD simulations (in the absence of additional forces). The time-averaged backbone RMSD ranges from 1.1 to 1.5 Å depending on the trajectory, a value close to that computed from the conventional MD simulation performed on the OF open crystal structure (1.3 Å). Moreover, the funnel radius profile shows that the transporter is in an OF open state in all relaxation trajectories (S4C Fig). The arginine substrate remains bound to the binding pocket in all simulations but two, where it is basically rotated from its crystal position, as shown by the distribution of its RMSD (Fig 3A). In most of the trajectories, the Arg side chain is engaged in a cation-π interaction with Trp293 (located at the bottom of the binding site) (Fig 3B) and its backbone forms direct and/or water-mediated H bonds with Asn22, Ile23, Ser26, Gly27, Trp202, and Ile205, most of which belong to the central unwound part of TM1 or TM6. Compared to the tMD simulations, the backbone of the substrate forms with residues of the binding pocket fewer but more persistent H bonds (direct rather than water-mediated), providing a better match with the OF open substrate-bound crystal structure (Fig 3B). This smaller number of interacting residues (Fig 3B versus Fig 2B) results mainly from reduced fluctuations of the arginine substrate in the binding site during the relaxation MD.

#### Occlusion of the OF open substrate-bound state

**Fig 4:**
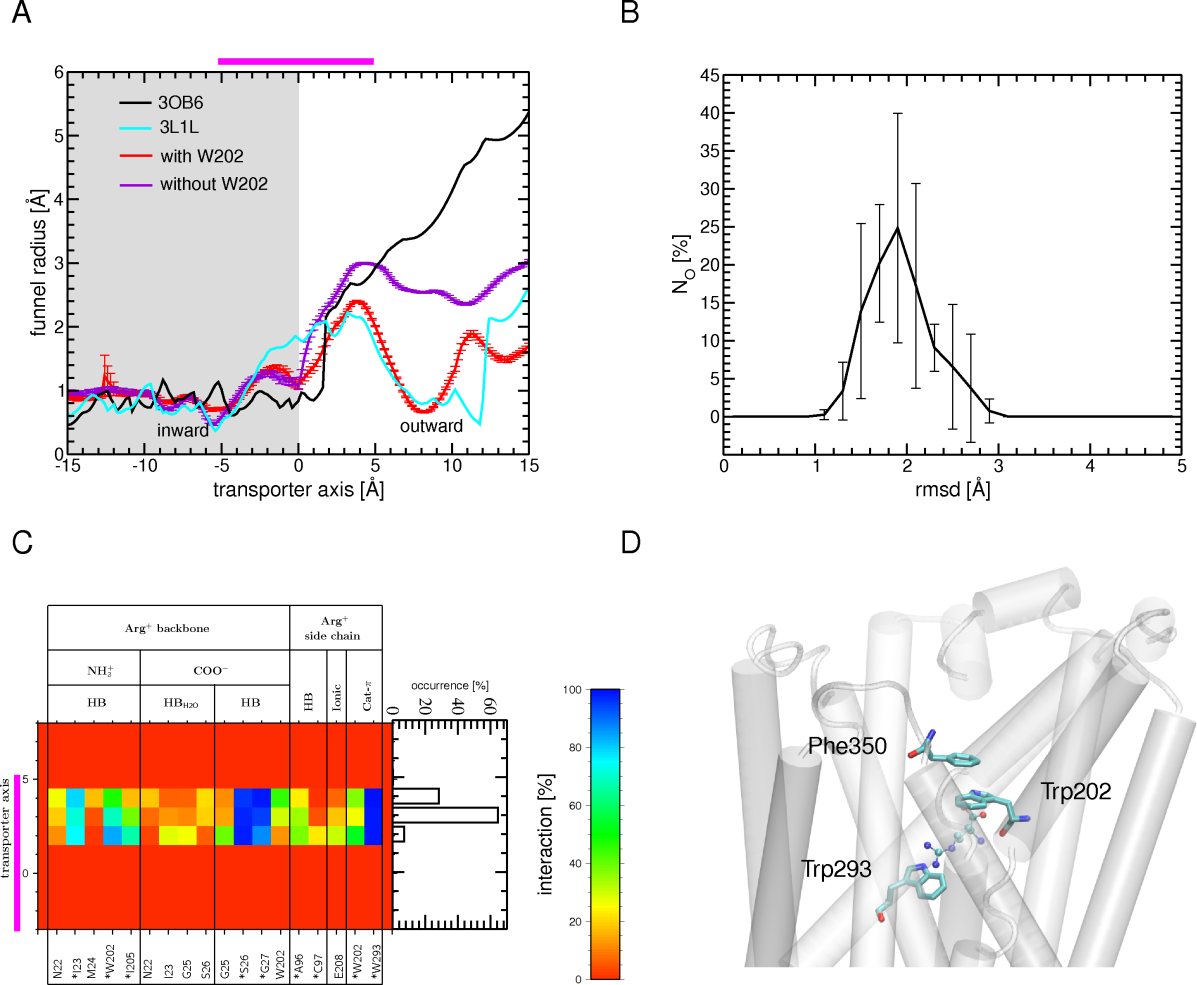
Transition from the OF open to the occluded substrate-bound state. (A) The profile of the funnel radius as a function of position along the main axis of the transporter, averaged over the last 0.1 ns of one tMD simulation, is shown with (red) and without (violet) the inclusion of Trp202 in the targeted ensemble of atoms (see text). The standard error is shown as bars. The radius is also depicted for the OF open (3OB6, black) and occluded (3L1L, cyan) substrate-bound crystal structures. (B) Number of occurrences (N_O_) for finding the substrate at a certain RMSD value, computed for the carbon atoms of Arg^+^ using all relaxation MD simulations (black) and its crystal position in the occluded state (PDB ID: 3L1L) as a reference. The standard error is shown as bars. (C) Interactions (H bonds, ionic and cation-π) formed, during all relaxation MD simulations, between the Arg^+^ backbone or side chain and protein residues. Only interactions with an occurrence higher than 20% in at least one bin width from all 12 binding events of the 6 tMD trajectories are shown. The interactions formed in the occluded substrate-bound crystal structure (PDB ID: 3L1L) are indicated by an asterisk. The abbreviations used for the different interactions are listed in the legend of Fig 2. (D) One typical position of Arg^+^ in the binding site is depicted. The aromatic residues Trp202 and Trp293, sandwiching the Arg^+^ side chain, and Phe350 and Trp202, making the π-π interaction formed upon OF occlusion are shown as sticks and the protein portion shaping the OF side is shown as a white cartoon. The substrate Arg^+^ is depicted as CPK. The binding site region (-5 to 5 Å) is highlighted by a magenta bar in (A) and (C).

To investigate the transition to the occluded state and to reveal the critical role of specific residues in this process, we performed tMD simulations starting from the last conformation of three of the six relaxation trajectories of the Arg^+^-bound OF open structure (Fig 1A: step 1b; S1 Table) and targeting backbone portions of several TM fragments in the occluded crystal structure of AdiC (Fig 1A: step 2a). The funnel opening shows that no complete occlusion of the OF side occurs in most of the tMD end conformations (Fig 4A). The monomers which remain open feature a rotameric state of Trp202 like that observed in the OF open crystal structure. In contrast, in the trajectories where occlusion occurs, Trp202 adopts a rotameric state similar to that observed in the occluded crystal structure. This finding prompted us to carry out other tMDs, starting from the last conformation of all six relaxation trajectories of the Arg^+^-bound OF open structure and including the Trp202 side chain among the targeted portions of the transporter, in addition to the protein segments selected for the previous tMDs (Fig 1A: step 2b; S1 Table). At the end of all six tMD simulations, the rotamer transition of the Trp202 side chain is achieved in 10 out of the 12 monomers allowing closure of the OF funnel as observed in the occluded crystal structure. In the other two monomers, an incomplete transition of the Trp202 side chain occurs, leading to only partial closure of the OF state. This result stresses that a particular rotameric state of Trp202 is crucial for occlusion of the OF open state, as suggested in previous theoretical and crystallographic studies [15,50]. At the end of the relaxation simulations performed after the tMDs, the transporter remains in its occluded conformation, as illustrated by the funnel opening profile (S5 Fig). In keeping with this observation, the time-averaged backbone RMSD of the protein ranges from 1.8 to 2.2 Å depending on the individual trajectory, a value fairly close to that (1.5 Å) computed from the 10-ns conventional MD simulation performed on the occluded crystal structure.

The arginine substrate, though not included in the targeted set of atoms, remains bound to the binding pocket in all 12 monomers, as shown by the distribution of its RMSD values (Fig 4B) and by the interactions it forms with the binding site residues (Fig 4C). By comparison with the OF open substrate-bound conformations, the Arg^+^ side chain forms one additional H bond with C97 and one cation-π interaction with Trp202 after occlusion has occurred (Fig 4C versus Fig 3B). The cation-π interaction with Trp202 is formed after transition of the aromatic side chain to its rotamer in the occluded crystal structure. This promotes a configuration where the ligand is sandwiched between two aromatic residues, Trp202 and Trp293, as observed in the AdiC occluded crystal structure [14]. Interestingly, this closure also brings Phe350 close to Trp202, so that they may form together a π-π interaction consolidating closure of the OF side (Fig 4D). Overall, most of the interactions made by arginine are identical to those observed in the occluded crystal structure [14] (Fig 4C).

#### Transition from the occluded to the IF open substrate-bound state

To allow release of the arginine substrate, the occluded state must undergo a transition to an IF state (Fig 1A). To the best of our knowledge, AdiC has not yet been trapped in this structural state. We thus simulated the transition from the occluded to the IF open substrate-bound state using the structure of GadC, another AR transporter [27]. Though this structure is substrate-free, its binding pocket is clogged by its small carboxy-terminal domain, referred to here to as the C-plug. This plug is proposed to block the transport path at pH values not requiring GadC transport activity and to be dislodged at acidic pH to allow transport [9,27].

Several residues conserved between AdiC and GadC are reported to be essential to the function of these two antiporters, suggesting that their transport mechanisms share common features [15,25–27]. It has been proposed that the IF open conformation of GadC, omitting the C-plug, corresponds to a distinct state of AdiC during substrate transport [27]. To model the transition to the IF substrate-bound state, we performed tMD simulations targeting the backbone atoms of several TM fragments selected on the basis of the more complete of the two available GadC structures (Fig 1A: step 3a or 3b) and starting from the last conformation of three different relaxation trajectories of the occluded structure (Fig 1A: step1b).

**Fig 5:**
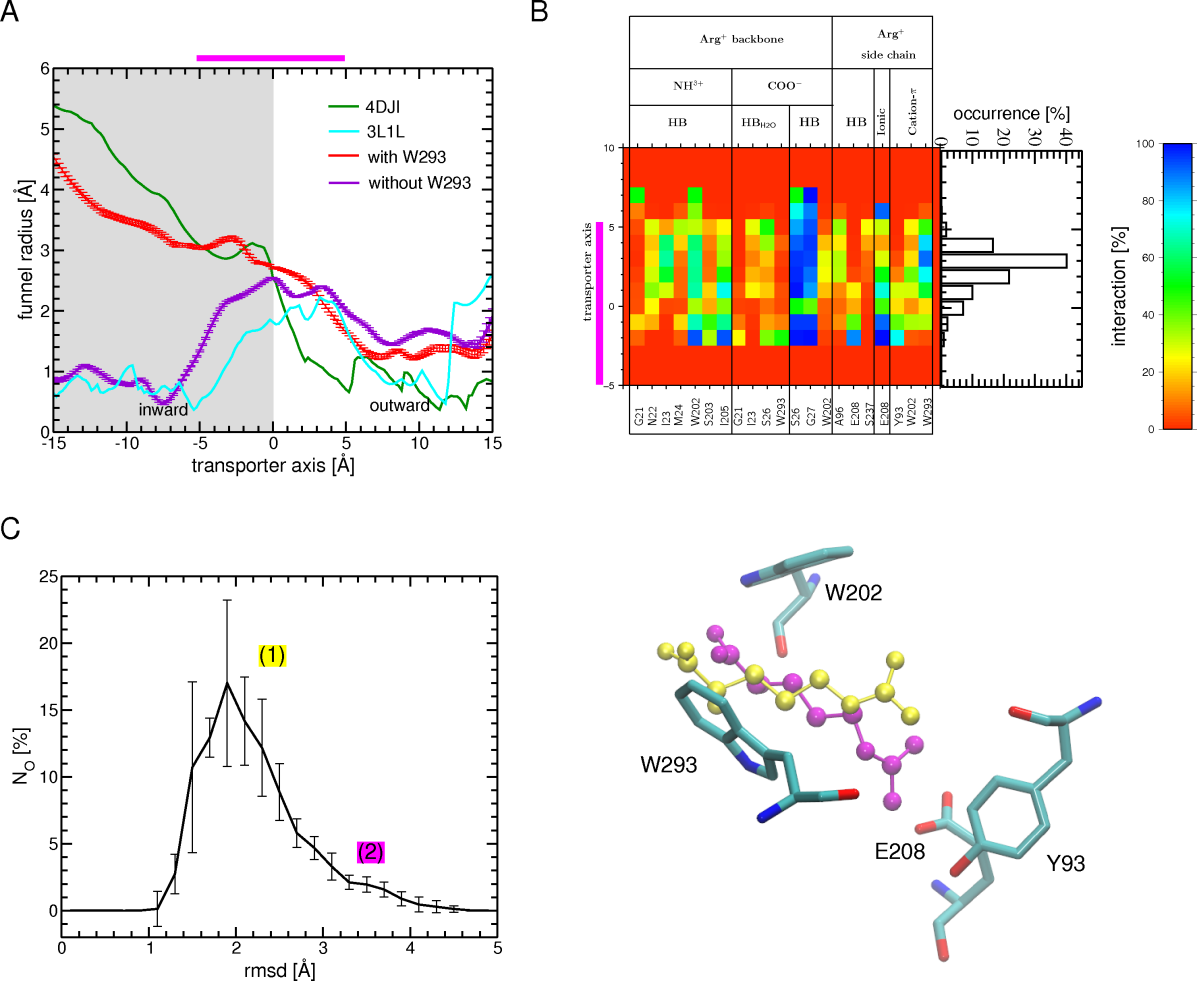
Transition from the occluded to the IF open substrate-bound state. (A) The profile of the funnel radius as a function of position along the main axis of the transporter, averaged over the last 0.1 ns of one tMD simulation is shown with (red) and without (violet) the inclusion of Trp293 in the targeted atom ensemble (see text). The standard error is shown as bars. The radius is also depicted for the IF open GadC (4DJI, green) and the occluded AdiC (3L1L, cyan) crystal structures. (B) Interactions (H bonds, ionic and cation-π) formed by the Arg^+^ backbone or side chain with protein residues during all tMD trajectories. Only interactions with an occurrence higher than 20% in at least one bin width from all 24 binding events of the 12 tMD trajectories are shown. The abbreviations used for the different interactions are listed in the legend of Fig 2. The binding site region (-5 to 5 Å) is highlighted by a magenta bar in (A) and (B). (C) (left) Number of occurrences (N_O_) for finding the substrate at a certain RMSD value, computed for the carbon atoms of Arg^+^ using all tMD simulations and its crystal position in the occluded state (PDB ID: 3L1L) as a reference (left). The standard error is shown as bars. Two different Arg^+^ positions (labelled (1) and (2)) are depicted. The position (1) (in yellow CPK) corresponds to the position with the highest occurrence and the (2) (in magenta CPK) features Arg^+^ with its sidechain reoriented towards Glu208. Surrounding binding site residues are shown as sticks (atom type colored). These two positions are marked with their number on the RMSD plot.

At the end of the three tMD simulations, none of the six monomers adopts a conformation close to an IF open state. The monomers remain, rather, in an occluded form, as shown by the funnel opening (Fig 5A). To tackle this problem we included the side chain of Trp293, which was proposed to form a gate liable to open the transporter to the IF state [14], among the regions targeted during the tMD simulations and used, in this case, both GadC structures, as they feature different rotamers of Trp308, the residue corresponding to Trp293 in AdiC. In line with this Trp293 was moreover proposed, based on an AdiC IF model, to move along with Arg substrate during the transition from the occluded to the inward side [15]. Moreover, its replacement by non-aromatic residues is reported to abolish arginine transport in AdiC, whereas a change to other aromatic residues (Phe, Tyr) causes only a reduction of transport [15]. Likewise, mutation of the corresponding residue in GadC (Trp308) to Ala abolishes Glu transport [27]. Remarkably, inclusion of Trp293 has a tremendous impact: the tMD simulations produce conformations featuring an IF open state, as shown by the funnel radius profile (Fig 5A).

The interactions of the arginine backbone remain almost identical to those observed in the occluded state (compare Fig 5B to Fig 4C). Towards the end of most tMD trajectories, the arginine side chain reorients itself towards the intracellular side (Fig 5C). This rearrangement produces interactions of Arg^+^ with Glu208 (ionic) and, unseen before this step, with Tyr93 (cation-π) (Fig 5B and 5C), at the expense of an H bond with Ala96 and of cation-π interactions with Trp202 and Trp293, formed in the occluded state and weakened or lost at this stage. These data suggest that Arg^+^ exits the binding pocket side chain first. Both Glu208 and Tyr93 have been proposed to form part of a gate between the binding site and the intracellular medium [25]. This distal gate, comprising Tyr365 in addition to the two residues just mentioned, was identified on the basis of a crystal structure and mutational studies [14,25,26]. In the occluded crystal structure, Glu208 does indeed form H bonds with both Tyr365 and Tyr93, establishing a gate between the binding site and the IF side of the transporter [14]. Opening to the IF side, promoted in part by displacement of TM6 (which includes Glu208; see Material and Methods), results in break-up of the layer formed by these three residues. Glu208 separates from Tyr93 while maintaining its H bond with Tyr365 (S6A Fig), allowing the arginine substrate to slide down towards the inside medium. No direct interactions between Tyr365 and the arginine substrate are formed in our simulations, suggesting a possible role for Tyr365 that is to stabilize the orientation of Glu208 in the opening towards the IF side.

In most of the subsequent relaxation simulations and regardless of the GadC structure used as target, a large number of the IF end conformations feature a significant closure of the inward funnel (S6B Fig), suggesting that these IF substrate-bound conformations are not stable. To investigate the possible causes of this instability the time evolution of the restraint potential of the occluded to IF open transition was calculated and compared for several TMDs trajectories, differing by their stability of their IF reached states (S6B Fig). This comparison shows no correlation between the TMD trajectories featuring a lower restraint potential and the capacity of the TMD end conformation to remain in an open stable state.

To examine further the instability issue of the IF reached states we constructed a curtailed Arg^+^ transport process by simulating the transition from the occluded to the IF substrate-bound state starting from the occluded crystal structure (PDB ID: 3L1L; S1A Fig: step 5; S2 Table). This was done to check whether the instability of the IF conformations was due to the preceding simulated steps (Fig 1A: step 1 to step 3). In these tMD simulations, the most persistent interactions of the arginine substrate with neighboring residues are similar to those observed in the simulations carried out starting from the OF open crystal structure, except for the interaction with Asn101, which is seen to engage in an H bond with the arginine side chain (S7A Fig versus S5B Fig). Also, the arginine substrate features a similar RMSD distribution, though narrower than in the simulations performed starting with the occluded crystal structure (S7B Fig versus Fig 5C). More importantly, a majority of the subsequent relaxation trajectories, albeit fewer than in the simulations starting from the OF open crystal structure, also feature closure of the IF conformations reached (S7C Fig).

Secondly, we assessed the stability of the IF state of GadC, exploiting simulations performed on one of its crystal structures (PDB ID 4DJI) [27] in the presence and absence of the C-plug. The rather low RMSD value recorded (1.5/1.6 Å; S8A and S8B Fig) and the radius of the inward-facing funnel (S8C Fig), which is as open as in the crystal structure, sustain that the GadC substrate-free crystal structure in contrast to the IF AdiC state is fairly stable along our simulations. This is not in support that the use of the GadC structure as the target structure is the cause of closure of the AdiC substrate-bound IF conformations although one cannot fully discard this possibility either.

It is also conceivable that an IF open substrate-bound state as such is intrinsically unstable. This possibility is raised by the observation that, among the crystal structures of APC-superfamily amino acid transporters determined so far, this state is less represented than the IF open substrate-free state (S2A Fig). To examine the stability of AdiC IF open substrate-free conformations, we performed MD simulations starting from the end conformation of the tMDs where arginine was removed (S4 Table). As shown by the funnel radius profiles (S9 Fig), the IF substrate-free state has a lesser propensity to close. This suggests that the IF substrate-free state is somehow more stable than the IF substrate-bound structure.

#### Release of Arg^+^ from the IF state

**Fig 6:**
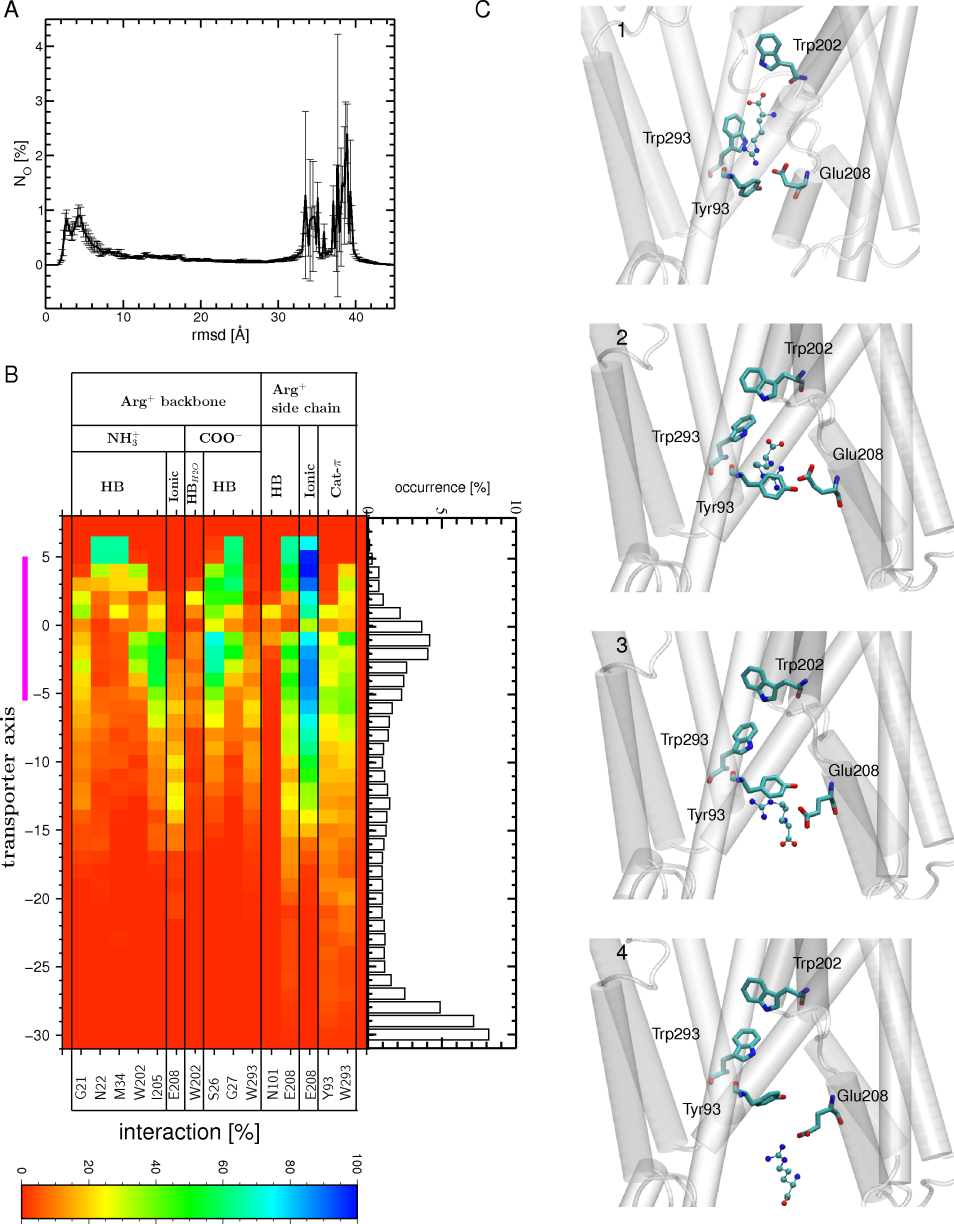
Release of arginine into the cytosol from the IF open state. (A) Number of occurrences (N_O_) for finding the substrate at a certain RMSD value, computed for the carbon atoms of Arg^+^ using all tMD simulations and its crystal position in the occluded state (PDB ID: 3L1L) as a reference. The standard error is shown as bars. (B) Interactions (H-bonds, ionic and cation-π) formed between the Arg^+^ backbone and side chain and protein residues during the tMD simulations. Only interactions with an occurrence higher than 20% in at least one bin width from all 72 binding events of the 36 tMD trajectories are shown. The abbreviations used for the different interactions are listed in the legend of Fig 3. The binding site region (-5 to 5 Å) is highlighted by a magenta bar. (C) Four tMD conformations highlighting the exit of arginine from the IF open state to the cytosol (1–4). The Arg^+^ side chain first leaves the primary binding site, still forming a cation-π interaction with Trp293, but from the IF side, in contrast to that formed in the OF state (Fig 5D), and making an ionic bond with Glu208 (1). The cation-π interaction with Trp293 is lost and the Arg^+^ side chain interacts mostly with two distal gate residues, Tyr93 (through a cation-π interaction) and Glu208 (2). The cation-π interaction with Tyr93 serves as a hinge to pivot the Arg^+^ side chain, which then forms an ionic bond to Glu208 via its amino group (3). The backbone heads first towards the exit, with the guanidinium group making an ionic interaction with Glu208 (4). Residues making persistent interactions with Arg^+^ are shown as sticks and the protein portion shaping the OF side is shown as a white cartoon. The substrate Arg^+^ is depicted as CPK.

**Fig 7:**
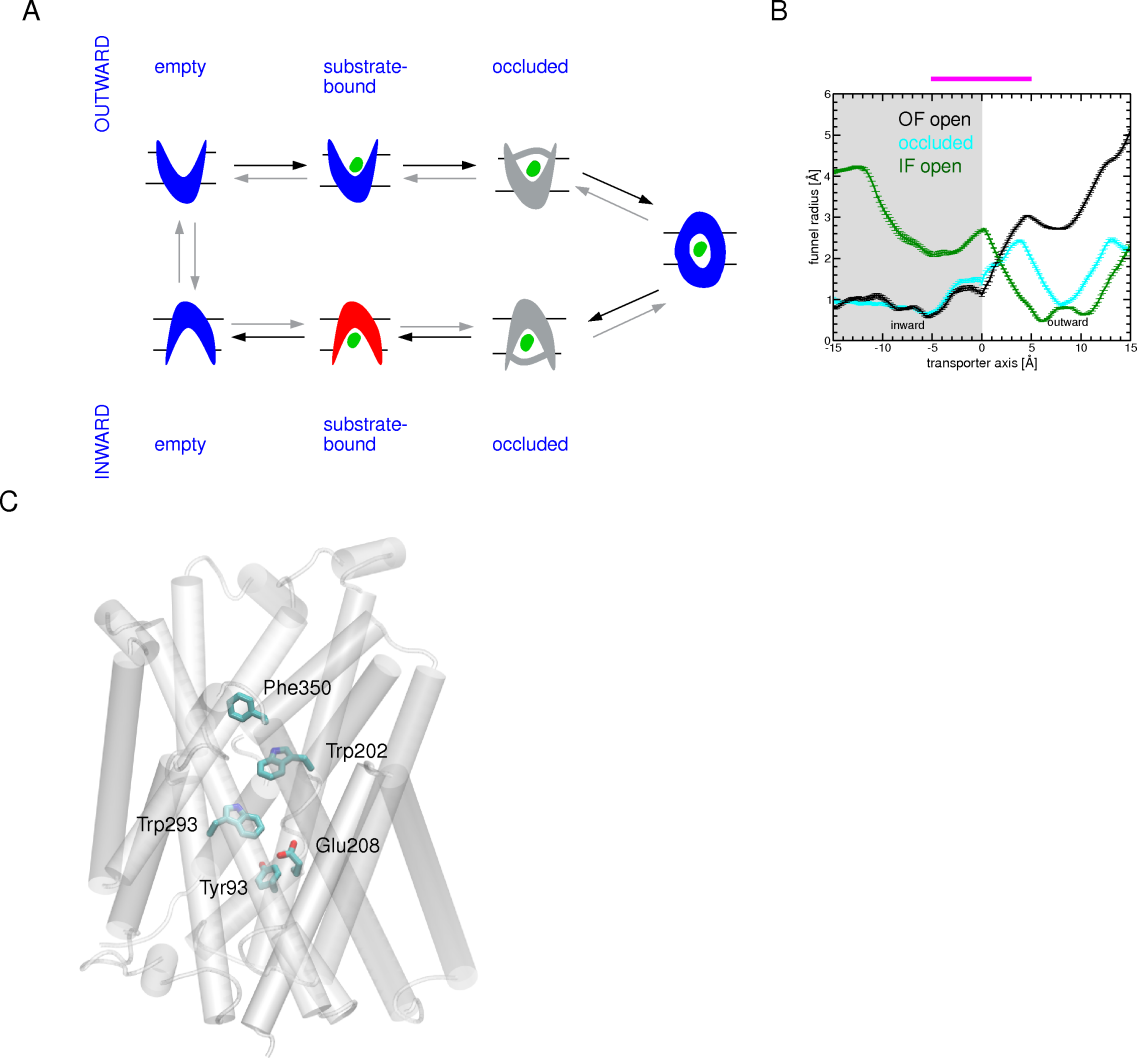
Illustration of the main outcomes of the simulated mechanism of arginine transport through AdiC. (A) Schematic representation of potential AdiC intermediate states along the Arg^+^ transport pathway, together with their funnel radius profiles. (Left) Structural states found to be populated along our simulations are highlighted in blue; the more likely unstable ones are colored in red. States that seem to be transient in the simulated transport are in gray. Black arrows indicate the migration direction of the arginine substrate. (Right) The funnel radius profile as a function of the position along the transporter axis, depicting the most stable structural states observed along the arginine translocation (OF open: black, occluded: cyan and IF open: green), averaged over the last 0.1 ns of one relaxation simulation in step 1, step 2b, and step 4 (Fig 2). The binding site region (-5 to 5 Å) is highlighted by a magenta bar. (B) Key residues assisting Arg^+^ migration along the simulated transport pathway. These residues are shown as sticks and the protein is shown as a white cartoon.

To promote the release of Arg^+^ towards the cytosol, tMD simulations were carried out. These simulations started from the end conformation of the tMDs featuring an open IF state at the end of step 3b (Fig 1A) and targeted arginine at randomly located positions in the intracellular medium (Fig 1A: step 4). The assumption underlying the use of these tMD conformations is that transition to an IF substrate-bound conformation should be immediately followed by a further migration of the arginine, without the occurrence of a stable intermediate IF open state with the substrate bound to the central binding pocket. The unbinding process could trigger side chain reorientations that would contribute to increasing the stability of the IF open substrate-free structure. Indeed, in contrast to the relaxation simulations performed on the IF open substrate-bound transporter (Fig 1A: step 3b) in which most though not all trajectories revealed a closure of the IF side of the transporter, various degrees of funnel opening on the IF side, i.e. quasi-open, semi-open, and closed, are observed in the relaxation MDs of the IF open substrate-free transporter, with about a third remaining in a stable IF open state (S10A Fig). This behavior is similar to that observed in simulations of the IF open state, which show that removal of Arg^+^ directly from its binding pocket leads to partial stabilization of the IF open state (S9 Fig). The distribution of the RMSD of arginine along the main axis of the transporter indicates that Arg^+^ dwells mainly at three locations (Fig 6A): (1) different positions spread outside the transporter in the cytosol, promoted by the targeted positions, (2) the primary binding site, as in the occluded crystal structure, and (3) a location such that the Arg^+^ backbone remains near the primary binding site, still forming H bonds with Gly21, Ser26, Gly27, Trp202, Ile205, and Trp293, whereas its side chain points towards the distal gate residues. In this position, the Arg^+^ side chain is stabilized by an ionic interaction with Glu208 and, to a lesser extent, by a cation-π interaction with Tyr93 or Trp293 (Fig 7B).

## Discussion

In this paper we have explored the transport of arginine by AdiC, an antiporter protecting *E*. *coli* against extreme acid stress. To simulate the translocation of arginine, we have performed a series of tMD and conventional simulations, using only three experimental crystallographic structures as guides. On the basis of their funnel radius values, these structures were characterized as adopting either an OF open, an intermediate fully occluded, or an IF open state. Transport was initiated from the OF open AdiC structure and routed through the other structures describing subsequent states along the transport pathway (Movie S1). One of the issues raised here is the role played in arginine migration by the dynamics of the different structural states and the specific interactions of protein residues with arginine. Analysis of the MD trajectories has led to identifying key residues assisting arginine translocation and/or contributing to major conformational changes. It has also revealed a potential molecular mechanism for arginine transport. The essential insights gained from this work are summarized and further discussed hereafter.

### Structural dynamics of arginine transport by AdiC

According to the alternating access paradigm, an APC transporter must pass through several distinct structural states in order to transport its substrate. Even though crystal structures are of tremendous importance in depicting states visited along the translocation pathway, they alone cannot ensure full understanding of the transport mechanism, as it is an inherently dynamic process. In addition, crystallographic structures may be altered by factors such as crystal contacts, and the use of detergents, and are sometimes determined for mutant proteins, which can blur a detailed understanding of transporter functional cycles [61]. Hence, our simulations, despite their limitations, provide insights into the conformational equilibrium of AdiC and into how arginine migration shifts this equilibrium to enable transport.

All three crystal structures (two AdiC structures and one GadC structure) incorporated as intermediate states to investigate the translocation pathway of arginine were chosen on the basis of their funnel radius profiles (S2 Fig). Both the conventional and relaxation MD trajectories show that these states are fairly well populated, with no high-probability transitions to other states. An exception is the IF open substrate-bound state, which appears unstable, as it returns to the fully occluded state in most though not all relaxation trajectories (S6B Fig). Our simulation data do not buttress however, that this instability arises from the successive simulation stages applied to the initial AdiC structure, from the use of GadC as the targeted structure, or even from targeting different rotameric states of Trp293 side chain, a residue crucial to opening on the IF side.

The existence of a likely transient arginine-bound IF state has been previously proposed [14]. It might be rationalized by the need for the transporter to rapidly displace arginine from its main binding site down to adopt possibly a different IF open substrate-bound state. In keeping with this idea of a transient IF substrate-bound state, a comparison of the OF occluded substrate-bound and IF substrate-free crystal structures of Mhp1 suggests that the substrate-bound IF state might be intrinsically unstable and might relax to an OF occluded conformation [11]. Likewise a survey analysis on secondary transport mechanism also proposes that the IF substrate-bound state is likely to be of higher energy relative to other conformational states [12]. Our relaxation MD simulations thus suggest that four states, OF open substrate-free, substrate-bound, fully occluded, and IF substrate-free, are potentially well populated. One state, IF substrate-bound, seems to be more transient, while the OF and IF substrate-bound occluded states are not observed. Even though the latter two states were not targeted in this study this does not rule out that they could not be visited along the translocation pathway in the event of a targeted unstable state.

Remarkably, this sequence of conformational states along the simulated arginine migration pathway which involves one fully occluded state (Fig 7A) corresponds fairly well with the recent cycle of major structural snapshots proposed for LeuT [40]. The conformational transitions either from the OF open to occluded or from the occluded to the IF open states are primarily achieved by driving a few TM portions, in particular TM1 and TM6.

### Key residues assisting arginine translocation

On its route from the OF side towards the IF side, Arg^+^ interacts persistently with only a few residues. Starting from the OF side, one aromatic residue, Phe350, forms interactions with the guanidinium group of arginine (Fig 2B and 2C) on its way towards the binding site. Arginine progression is subsequently facilitated by a cation-π interaction made with Trp202. Interestingly Trp202 and Phe350 formed a π-π stacking in an OF AdiC crystal structure [25] and a MD study suggested that this stacking occurs only in the presence of Arg in the binding site and could be a first step towards the occlusion [15].

In our simulations the two aromatic residues Phe350 and Trp202 seem to act in a complementary way to move arginine forward to its binding site. After its entry into the binding pocket, the arginine side chain is stabilized by another cation-π interaction persistently formed with Trp293. Consistently with the role of Trp202 and Trp293 in arginine recognition, replacement of Trp202 and Trp293 with Leu has been shown to strongly affect arginine uptake *in vitro* [15,26]. To the best of our knowledge the impact of Phe350 mutations on AdiC functional properties has not been described so far. We observed during the binding process of Arg to the OF open state that Phe350 at the entrance of the transporter assists Arg migration to the binding site by forming a cation-π interaction. In that respect the substitution of Phe350 into a non-aromatic residue could affect the K_M_ of AdiC. However, as suggested previously [15,25], we also found that closure of the OF open substrate-bound state brings Phe350 close to Trp202 leading to a π-π interaction. In this regard substitution of Phe350 into a non aromatic residue might affect V_max_.

The trajectories simulating occlusion of the OF conformation suggest that one rotameric state of Trp202 is of utmost importance: when it is not included among the protein portions targeted in the tMDs, along with the portions of TM1 and TM6 known to undergo the most significant displacements upon occlusion, no stable occluded state is reached (Fig 4A) unless a spontaneous rotation of Trp202 occurs during the tMD trajectories. Only with inclusion of the Trp202 side chain among the targeted protein portions is the occluded state reproducibly achieved. In this state, the arginine substrate is sandwiched, in the binding site, between two aromatic residues, Trp202 on the OF side and Trp293 on the IF side (Fig 4D). To reach this configuration, Trp202 undergoes a large displacement, resulting largely from movement of the TM6 backbone, and an internal rotation of its side chain, needed to achieve proper positioning enabling full occlusion of the binding site and formation of specific interactions with the arginine substrate. Notably, 2 out of the 3 aromatic residues involved in the migration of arginine and in forming the sandwich configuration are strictly conserved (Trp202, Phe350) in the yeast arginine transporters [62–64] ((S6 Table). Conservation of the third aromatic residue, Trp293, in the latter transporters is visible only when their modeled 3D structure is superimposed onto that of AdiC [62]. Upon occlusion, Phe350 and Trp202 form a π-π stacking whose occurrence is likely to strengthen closure of the binding site on the OF side (Fig 4D). Interestingly, a previous study has suggested that this particular π-π stacking is formed upon closure of the AdiC OF funnel [15,25]. To achieve the conformational transition from the occluded to the IF open state, reaching a specific Trp293 rotameric state is crucial (Fig 5A). In the generated IF conformations, novel persistent interactions of the arginine side chain are formed with two residues of the distal gate, in particular an electrostatic interaction with Glu208 and a cation-π interaction with Tyr93. These interactions are formed as Tyr93 separates from Glu208, thereby breaking the H bond between them (S6A Fig). At the same time the H bond between Glu208 and another distal gate residue Tyr365 is maintained (S6A Fig). Altogether this appears to contribute to an opening of the distal gate between Tyr93 and Glu208. Consistently with these observations, substitution of Tyr93 and Glu208 has been shown to strongly impact transporter affinity for or transport of arginine and/or agmatine [25].

The last step of the translocation shows that unbinding of arginine from its central pocket is initially driven by cation-π interactions of arginine with Trp293 and Tyr93 and by an ionic interaction with Glu208, facilitating migration of arginine towards the cytosol. In parallel Tyr93, via a cation-π interaction with the Arg^+^ side chain, acts as a pivot allowing Arg^+^ to flip and exit, backbone first, in most of the simulations.

In summary, there exist similarities between Arg^+^ migration along the OF side and the IF side. Upon entry and exit, Arg^+^ interacts persistently with one aromatic residue, Phe350 (OF side) or Tyr93 (IF side) (Fig 7B). The major binding site is delineated by two aromatic residues Trp202 (OF side) and Trp293 (IF side) (Fig 7B) which sandwich the substrate. Furthermore, the substrate enters the transporter side chain first and exits it side chain last. This points to the guanidinium moiety as the main driving group in essential steps of the translocation.

Overall, our study reveals important coupling between the motions of the protein and those of arginine in assisting migration of the latter. Furthermore, our trajectories clearly support the view that local conformational changes, in addition to global structural changes of a few TMs, are fundamental to transitions between states. These findings confirm the existence of dynamic “gates” that can open and close to allow alternate access of the ligand to either side of the transporter. We find that these local changes mainly involve interconversion between rotamers of the residues forming these gates. Arginine is grasped first by Phe350 and subsequently by Trp202 (proximal gate), whose side-chain rotation occludes the OF side and serves as the proximal gate. This closure is strengthened by a π-π interaction between Phe350 and Trp202. Phe350 might thus possibly form an upper doorway preceding the proximal “Trp202 gate”. Arginine is released on the other side, assisted by the rotamer transition of another tryptophan (Trp293) forming an inner (middle) gate. Furthermore, during the transition from the occluded to the IF state, the distal gate opens as residues Tyr93 and Glu208 separate from each other. These two residues then act together with Trp293 to pull arginine out towards the cytosol. Overall, our simulations clearly highlight that the middle and distal flexible gates do not work independently of each other during the transitions.

### Transport mechanism features shared by the AR transporters

Strikingly, most of the residues found to interact persistently with Arg^+^ along its translocation through AdiC are aromatic. Given the chemical nature of Arg^+^, the first question that comes to mind is whether cation-π interactions play a key role in the translocation process. Arg^+^ contains two groups capable of forming such interactions, namely its backbone amino and side-chain guanidinium groups. Our simulations point to a stronger involvement of the arginine side chain in forming specific interactions with different aromatic side chains along the translocation path.

This observation prompted us to look into the conservation of Phe350, Trp202, Trp293, and Tyr93 in AR transporters. All are strictly conserved in the *E*.*coli* ornithine/putrescine and lysine/cadaverine antiporters, the side chain of whose substrate can also form cation-π interactions (S6 Table). The involvement of mainly aromatic residues —and not of negatively charged residues —in the transport of positively charged amino acid and amine analogs has been explained by the acidic environment faced by AR transporters, which likely protonates acidic side chain residues so that they can no longer form salt bridges [26].

GadC, which exchanges a negatively charged Glu^-^ or a neutral Glu^0^ (having a protonated side chain carboxyl group) for GABA^+^ [27,65], also possesses aromatic residues at the positions corresponding to Phe350, Trp293, and Tyr93 but not at that corresponding to Trp202, where there is a leucine (Leu212) (S6 Table). The conservation of Trp202 in AdiC, PotE, and CadB only stresses the essential role of an aromatic residue at this position in leading to and stabilizing in the binding site, through formation of cation-π interactions, amino acids with positively charged side chains entering from the OF state. The conservation of the other three aromatic residues in all four AR transporters raises the question of whether they play a role in translocating their substrates through formation of cation-π interactions. Yet Glu and GABA^+^ both possess a positively charged amino group, more accessible in the former than in the latter, which might form during substrate translocation a cation-π interaction with the aromatic side chains mentioned in this study (or their counterparts). In support of this hypothesis, the importance of cation-π interactions has been reported in the binding of GABA and even that of Glu to neurotransmitter receptors [66–68]. Overall, all three of the aromatic residues conserved in the four AR transporters could also serve as key elements in export of the decarboxylated forms of the amino acids: agmatine, putrescine, cadaverine, and GABA.

In addition to the four aromatic residues, Glu208 plays an important role, via formation of an ionic bond with its side chain, in releasing Arg^+^ on the IF side of the transporter. As for the pivotal aromatic residues, this acidic residue is strictly conserved in all four AR antiporters, raising the possibility of another role for this residue in GadC. Interestingly, the findings of one MD study suggest that the protonation state of Glu208 in AdiC could cause the release of Agm^2+^ but not of Arg^+^ on the outward-facing side [50]. Glu218 in GadC might play a similar role in the translocation of GABA^+^. The knowledge gained from this study suggests that the AR transporters, including GadC, share several mechanistic features, but also that key residues may play different roles during the import and export of substrates.

In conclusion, our MD data clearly highlights the importance of a few protein residues both in substrate binding and in the conformational changes accompanying the transport of arginine through AdiC. These residues are strongly conserved in the AR antiporters and in arginine permeases featuring the same structural fold. This strengthens the idea that these transporters share a common global transport mechanism with, however, distinct particularities that have evolved to adapt to the transported substrate.

## Supporting Information

S1 Movie Transport of arginine through AdiC. The successive steps of the migration of arginine describing the transport cycle are shown on the left. A selected single trajectory depicting each step is illustrated on the right. The protein is shown as a white cartoon and the residues found to be essential for the transport are depicted as sticks. The Arg^+^ substrate is shown as van-der-Waals spheres. The position of the binding site is highlighted with an orange frame.
(MP4)

S1 File Detailed information on Material and Methods. (DOCX)

S1 Fig Schematic representation of the arginine transport by AdiC antiporter and its reconstruction by MD simulations. (A) Reconstruction of truncated arginine transport by AdiC starting from the AdiC occluded substrate-bound structure using tMDs followed by relaxation MD simulations: (Step 5) Transition from the occluded substrate-bound to the IF open substrate-bound state targeting each of the two IF open crystal structures of GadC (PDB IDs: 4DJI or 4DJK [27]) including Trp293 side chain in the targeted protein portions (for further details see Material and Methods and S2 Table). (Step 6) Release of the arginine substrate starting from the final conformation of the tMDs obtained from step 5. Black arrows indicate the migration direction of the arginine substrate. (B) Conventional MD simulations performed on AdiC and GadC crystal structures adopting different conformational states: the AdiC OF open arginine-bound structure without and with the arginine substrate (simulation A and B, respectively), the occluded arginine-bound structure (simulation C) and the GadC IF open substrate-free structure with (simulation D1) and without its C-plug (simulation D2) (for further details see Material and Methods and S3 Table).
(TIFF)

S2 Fig Transport cycle of amino acid transporters of the APC superfamily and their funnel radius profile. (A) A schematic representation of the transport cycle featuring a series of potential conformational states. Substrate (green ellipsoid) binding to the OF open state promotes occlusion of the transporter. A potential OF occluded state undergoes a subsequent conformational change that switches it into an IF occluded state. Opening of the binding site towards the IF side leads to the release of the substrate. Other intermediate states may be sampled along the transport cycle such as the IF occluded substrate free state that was proposed to be adopted by ApcT (PDB ID: 3GI8 [33]). In antiporters, the return from the IF open to the OF open unbound structure requires the binding and transport of a second substrate but in the opposite direction. Each of the potential conformational states is exemplified by crystal structures (identified with their PDB IDs) either of the AdiC and GadC AR antiporters [14,15,25–27], LeuT [16,30] or MhsT [23] and taken from published reports. Black arrows indicate the migration direction of the arginine substrate. (B-D) The profile of the funnel radius, computed for crystal structures of amino acid transporters belonging to the APC superfamily selected from panel A, as a function of position along their main axis. Panel D combines both the OF and IF occluded states and also includes the funnel radius profile computed for ApcT used as an example of an IF occluded substrate-free structure. The binding site region (-5 to 5 Å) is highlighted by a magenta bar in (B-D).
(TIFF)

S3 Fig Structural and dynamic properties computed from a 10-ns conventional MD simulation of the occluded and OF open substrate-bound crystal structures of AdiC. The latter was performed after removal of the arginine ligand. (A) Time evolution of the backbone RMSD of the simulations of the occluded structure (S1B Fig: simulation C). (B) Backbone positional RSMF of the monomer A (blue) and monomer B (red) of the trajectory of the occluded structure. (B) The profile of the funnel radius as a function of the position along the main axis of the transporter, averaged over the last 0.1 ns of the 10-ns conventional simulation of the occluded structure (orange). For comparison the profile is also depicted for the occluded crystal structure (3L1L, cyan). (D) Time evolution of the backbone RMSD of the simulations of the OF open state (S1B Fig: simulation A). (E) Backbone positional RSMF of the monomer A (blue) and monomer B (red) of the trajectory of the OF open structure. (F) The profile of the funnel radius as a function of position along the main axis of the transporter, averaged over the last 0.1 ns of the conventional simulation of the OF open structure (orange). For comparison the profile is also depicted for the OF open crystal structure (3OB6, black). In B and D transmembrane helical regions are highlighted by grey stripes. In E and F the standard errors are shown as orange bars. The simulation of the OF open substrate-bound structure was performed after removal of the arginine ligand. The binding site region (-5 to 5 Å) is highlighted by a magenta bar in (C) and (F).
(TIFF)

S4 Fig The binding process of Arg^+^ to the OF open state. (A) Arginine binding pathways observed in the twelve different monomers from the six tMDs are depicted by spheres marking the positions of the C_β_ atom of Arg^+^ using snapshots extracted every 50 ps. The protein is shown as a white cartoon. Each ligand trajectory is colored differently. (B) Reorientation of Phe350 during the MD trajectory as shown by its starting position (1) in the AdiC OF open substrate-bound crystal structure (PDB ID: 3OB6 [15]) and at the end of the MD simulation (3). The location of Phe350 in the OF open substrate-free crystal structure (2) (PDB ID: 3LRB [25]) is also shown as a reference. Phe350 is depicted as sticks and the protein as white cartoon. (C) The profile of the funnel radius as a function of the position along the main axis of the transporter for one of the twelve monomers in the six tMDs of the dimers averaged over the last 0.1 ns of the relaxation trajectory (red). For comparison the profile is also depicted for the OF open crystal structure (3OB6, black). The standard errors are shown as red bars. The binding site region (-5 to 5 Å) is highlighted by a magenta bar.
(TIFF)

S5 Fig Transition from the OF open to the occluded substrate-bound state. The profile of the funnel radius as a function of the position of the main axis of the transporter, averaged over the last 0.1 ns of one tMD (blue) and of its subsequent relaxation (red) simulations including Trp202 in the targeted ensemble of atoms (see main text). The standard errors are shown as bars. The radius profile is also depicted for the OF open (3OB6, black) and occluded (3L1L, cyan) substrate-bound crystal structures. The binding site region (-5 to 5 Å) is highlighted by a magenta bar.
(TIFF)

S6 Fig Transition from the occluded to the IF open substrate-bound state. (A) Evolution of the distance between the Cδ atom of Glu208 and either the Tyr93 (blue) or Tyr365 (red) side chain hydroxyl oxygen atom averaged over the tMD trajectories. (B) The profile of the funnel radius as a function of the position along the main axis of the transporter, averaged over the last 0.1 ns of one tMD (blue) and of its subsequent relaxation MD (red) simulations. The standard errors are shown as bars. The profile is also depicted for the occluded AdiC (3L1L, cyan) and the IF open (4DIJ, green) GadC crystal structure. The binding site region (-5 to 5 Å) is highlighted by a magenta bar. (C) The restraint potential U_TMD_ is shown for three different trajectories (blue, red, and yellow curves) as a function of the tMD simulation time. An inlay shows a close-up of the curve between 0 and 0.6 kcal/mol. Vertical peaks occur when the instantaneous best-fit RMSD of the current coordinates are quite different from the target coordinates leading to a high restrained potential. The different curves correspond to a simulation in which, during the relaxation MD following the TMD, i) the two monomers close (yellow), ii) one monomer closes and the other remains open (blue) and iii) both monomers remain open (red).
(TIFF)

S7 Fig Transition from the occluded to the IF open substrate-bound state starting from the occluded crystal structure. (A) Interactions (H bonds, ionic and cation-π) formed between the Arg^+^ backbone and side chain and protein residues during the tMDs. Only interactions with an occurrence higher than 20% in at least one bin width from all 12 binding events of the 6 tMD trajectories are shown. The abbreviations used for the different interactions are listed in the legend of Fig 3. The binding site region (-5 to 5 Å) is highlighted by a magenta bar. (B) Number of occurrences (N_O_) for finding the substrate at a certain RMSD value computed for the carbon atoms of Arg^+^ using all tMD simulations and its crystal position in 3L1L as a reference. The standard errors are shown as bars. The RMSD values corresponding to the two representative positions of Arg^+^ shown in Fig 6C are numbered accordingly. (C) The profile of the funnel radius as a function of the position along the main axis of the transporter, averaged over the last 0.1 ns of one tMD (blue) and of its following relaxation simulation (red). The standard errors are shown as bars. The profile is also depicted for the IF open GadC (4DJI, green) and the OF occluded AdiC crystal structure (3L1L, cyan).
(TIFF)

S8 Fig MD simulations of the GadC IF open state. (A-B) Time evolution of the backbone RMSD of the 10-ns long GadC simulations (A) with and (B) without the plug. (C) The profile of the funnel radius as a function of the position along the main axis of the transporter (PDB ID: 4DJI [27]), averaged over the last 0.1 ns of the MD simulations with (blue) or without (magenta) the C-plug. The standard errors are shown as bars. The profile is also depicted for the GadC IF crystal structure (4DJI, green). The binding site region (-5 to 5 Å) is highlighted by a magenta bar.
(TIFF)

S9 Fig MD simulations starting from the end conformations of the tMDs featuring an IF state and after removal of the arginine. The profile of the funnel radius as a function of the position of the main axis of the transporter, averaged over the last 0.1 ns of one tMD simulation (violet). The standard errors are shown as bars. For the sake of comparison, the profile is also depicted averaged over the last 0.1 ns of one of the relaxation trajectories (red) simulating the transition from the occluded to IF open Arg^+^-bound states (Fig 2A: step3b) as well as for the IF open GadC (4DJI, green) and the OF occluded AdiC (3L1L, cyan) crystal structure. The binding site region (-5 to 5 Å) is highlighted by a magenta bar.
(TIFF)

S10 Fig Release of the arginine substrate to the cytosol from the IF open state. (A) The profile of the funnel radius as a function of the position along the main axis of the transporter, averaged over the last 0.1 ns of different relaxation simulations features either an IF quasi-open (violet), semi-open (brown) or closed (red) state. The standard errors are shown as bars. For the sake of comparison, the profile is also depicted for the IF open GadC (4DJI, green) and the OF occluded AdiC (3L1L, cyan) crystal structure. The binding site region (-5 to 5 Å) is highlighted by a magenta bar. (B) Arginine release pathways in the 72 different monomers during the 36 tMDs are depicted by spheres marking the positions of the C_β_ atom of the ligand using snapshots extracted every 50 ps from the tMD trajectories. The protein is shown as a white cartoon. Each ligand trajectory is colored differently.
(TIFF)

S1 Table Overview of the simulations performed to model the transport of Arg^+^ starting from the OF open structure. Simulated steps are numbered according to Fig 2 (see main text). The simulations started with the binding of Arg^+^ to the OF open structure using two different initial positions (Conf1, Conf2) of the protein extracted from a classical MD simulation of the OF open crystal structure after removal of the ligand (S1B Fig: simulation B) and three different starting positions for Arg in the external medium leading to a total of 6 simulations for this step. Either classical MD (step 1a) or tMD followed by relaxation MD simulations (step 1b) were performed. The occlusion was modeled targeting the occluded substrate-bound AdiC crystal structure including (step 2b) or not (step 2a) W202 in the targeted ensemble of atoms. The transition to the IF open state was first simulated using the GadC crystal structure 4DJI as a guide (step 3a) (see Material and Methods for detail). A second series of simulations exploited protein portions from either of the two GadC crystal structures (4DJI or 4DJK) and included in both simulations W293 in the targeted ensemble of atoms (see Material and Methods) leading to a total of 24 simulations. The release of Arg^+^ to the cytosol was simulated starting from the last conformation of the tMDs produced from step 3b and three different positions of the arginine located in the cytosol were targeted leading to an ensemble of 72 simulations.
(DOCX)

S2 Table Overview of the simulations performed to model the truncated transport of arginine starting from the occluded substrate-bound state. The simulations are summarized in S1A Fig. Three different conformations of the AdiC-Arg^+^ complex (Conf1, Conf2, Conf3) were extracted from a classical MD simulation of the occluded substrate-bound AdiC crystal structure (S1B Fig: simulation C; S3 Table). For the transition from the occluded to the IF open state (step 5) protein portions from either of the two GadC crystal structures (4DJI or 4DJK) were targeted (for more details see Material and Methods) leading to 6 different simulations. For the simulations of the Arg^+^ release to the IF side (step 6) three different positions of the arginine located in the cytosol were targeted leading to a total of 18 simulations for this step.
(DOCX)

S3 Table Classical simulations of the AdiC and GadC crystal structures adopting different conformational states. (DOCX)

S4 Table Overview of the classical simulations performed starting from the final conformation of the tMDs simulating the occluded to the IF open transition in AdiC. The simulations were performed after removal of arginine and filling the binding site with water molecules (Fig 2A: step 3b).
(DOCX)

S5 Table The different types of interactions formed between Arg^+^ and protein residues monitored along the different trajectories. The electrostatic interactions have been calculated using vmd [57]. All other interactions were identified using eucb [59]. The abbreviations used for the different interactions in this table and different figures are: ionic for ionic interaction, HB for hydrogen bond, HB_H2O_ for water-mediated hydrogen bond, and Cat-π for cation π interaction.
(DOCX)

S6 Table Conservation of AdiC amino acids important for arginine translocation with E. coli AR antiporters (PotE, CadB and GadC) and with Can1, a representative of the arginine yeast transporters. (DOCX)
