# Supplementary material for: Unveiling the Mechanism of Arginine Transport through AdiC with Molecular Dynamics Simulations: The Guiding Role of Aromatic Residues
Source: PLoS One. 2016 Aug 2;11(8):e0160219. doi: 10.1371/journal.pone.0160219 (PMC4970712; doi:10.1371/journal.pone.0160219)
Supplement: S1 File — (DOCX) [file pone.0160219.s002.docx]

Title: Unveiling the mechanism of arginine transport through AdiC with molecular dynamics simulations: the guiding role of aromatic residues

-Supporting Information -

Short Title: Mechanism of arginine transport through AdiC

Authors: Eva-Maria Krammer^1*^, Kassem Ghaddar^1,2^, Bruno André^2^ and Martine Prévost^1*^

Affiliation

^1^Structure et Fonction des Membranes Biologiques, Université Libre de Bruxelles (ULB), Brussels, Belgium.

^2^Molecular Physiology of the Cell, Université Libre de Bruxelles (ULB), IBMM, Gosselies, Belgium

* Corresponding Authors

Email: ekrammer@ulb.ac.be (EMK), mprevost@ulb.ac.be (MP)

**Materials and Methods**

**Preparation of the transporter structures**

The missing loop (residues 345-350) in monomer A of the OF open arginine-bound structure of AdiC (PDB ID: 3OB6) [1] was constructed with MODELLER [2], using the loop in monomer B as template. The missing loop (residues 181-191) of the occluded arginine-bound structure of AdiC (PDB ID: 3L1L) [3] was built with MODELLER [2], taking the loop in monomer A of the OF open arginine-bound structure structure (PDB ID: 3OB6) as template. The missing loops (residues 184-186 and 469-472 in monomer A and 184-185, 398-402, and 478-474 in monomer B) of the more complete of the two available GadC IF open structures (PDB ID: 4DIJ [4]) were modeled either using the loops of the other monomer as template or *ab initio* with MODELLER [2]. All structures were oriented according to the OMP database [5] and hydrogen atom positions were added using the CHARMM GUI web interface [6]. For the structures determined for variant proteins, the mutated residues were substituted for the corresponding wild-type residue using the Mutate module of VMD [7].

The protonation probabilities of all titratable groups in all AdiC crystal structures were computed using the program MEAD/GMCT [8]. According to these predictions obtained at pH 5, all ionizable groups including Glu208, located near the binding site, were set at their standard protonation states in AdiC. Standard protonation states were also used for all titratable groups in GadC.

**Molecular dynamics simulation details and protocols**

All MD simulations were carried out on dimers of the transporters because of their physiological relevance and/or of the existence of a crystallographic dimeric assembly [1,9,10,4,11]. They were performed in the isothermal-isobaric ensembles at 300 K with the program NAMD2.9 [12]. The CHARMM27 force field [13,14] with CMAP corrections [15] was used to describe protein, water and ion atoms. A united atom force field described the lipids [16]. Long-range electrostatic interactions were calculated using the particle-mesh Ewald method [17]. A smoothing function was applied to truncate short-range electrostatic interactions. The Verlet-I/r-RESPA multiple time-step propagator [18] was used to integrate the equation of motion with a time step of 2 and 4 fs for short- and long-range forces, respectively. All bonds involving hydrogen atoms were constrained using the Rattle algorithm [19]. In all systems the KCl concentration was set at 0.1 M and the net positive charge was neutralized by addition of chloride ions. In total, each molecular system comprised ~110000 atoms, including about 360 lipid and 24000 water molecules. The box size was about 120×100×100 Å^3^.

**Simulations of AdiC and GadC crystal structures**

Classical MD simulations were carried out, using the following crystal structures: AdiC in the OF open substrate-bound state (with and without its arginine ligand), AdiC in the occluded substrate-bound state, and GadC in the IF open state, with or without its C-terminal plug (S1B Fig, S3 Table).

The OF open substrate-free AdiC molecular system was built starting from the dimer of the OF open arginine-bound N101A mutant structure (PDB ID: 3OB6) [1], after removal of the arginine substrate. The dimer was inserted into a hydrated 1-palmitoyl-2-oleoyl-sn-glycero-3-phosphoethanolamine lipid bilayer with the help of the CHARMM-GUI web server (http:/www.charmm-gui.org) [6]. The system was equilibrated for 35 ns with the fixed protein to remove possible clashes between the protein and its environment without altering the protein structure. The pre-equilibrated solvated bilayer was used to insert the other crystal structures generating the initial molecular systems (see below). The structure was further equilibrated for 20 ns with only the protein backbone fixed to relax the side chain atoms. Lastly, an unrestrained equilibration was performed for 20 ns. After equilibration, the Mutate module of VMD [53] was used to substitute Ala101 for Asn, the residue at this position in the wild-type transporter. The system was equilibrated for another 5 ns and a 10-ns production trajectory was generated. A similar protocol was applied to this OF open crystal structure in the presence of its bound arginine substrate.

The initial configuration of the occluded AdiC state was obtained by embedding the generated dimeric assembly of the occluded crystal structure (PDB ID: 3L1L) [3] in the pre-equilibrated bilayer-water system generated for the OF open configuration. Prior to protein equilibration, the Mutate module of VMD [7] was used to substitute Ala22 and Trp123, respectively, for the corresponding wild-type residues Asn and Leu. The system was equilibrated following the same protocol as for the OF open AdiC configuration. A 10-ns MD production run was generated.

Simulation was performed with the more complete of the two available GadC IF open structures (PDB ID: 4DIJ). The crystallographic GadC dimer was inserted into the pre-equilibrated bilayer-water system (see above). Another simulation was carried out, using GadC without its C-plug spanning residues 470 to 502. The groove left empty upon removal of this part was carefully hydrated. Both GadC systems were equilibrated as for the other AdiC systems. 20-ns production simulations were generated.

Conventional MD simulations were also carried out starting from the final configuration of the tMDs performed in step 3b (Fig. 2A; S4 Table). After removal of the arginine substrate and solvation by water molecules, the system was equilibrated for 5 ns with the protein kept fixed. This was followed by a 10-ns production run.

**References**

1. Kowalczyk L, Ratera M, Paladino A, Bartoccioni P, Errasti-Murugarren E, et al. (2011) Molecular basis of substrate-induced permeation by an amino acid antiporter. Proc Natl Acad Sci U S A 108: 3935–3940. Available: http://www.pubmedcentral.nih.gov/articlerender.fcgi?artid=3054010&tool=pmcentrez&rendertype=abstract.

2. Eswar N, Webb B, Marti-Renom MA, Madhusudhan MS, Eramian D, et al. (2006) Comparative Protein Structure Modeling Using Modeller. Current Protocols in Bioinformatics. Hoboken, NJ, USA: John Wiley & Sons, Inc., Vol. 5. pp. 5.6.1–5.6.30. Available: http://www.ncbi.nlm.nih.gov/pubmed/18428767. Accessed 22 August 2014.

3. Gao X, Zhou L, Jiao X, Lu F, Yan C, et al. (2010) Mechanism of substrate recognition and transport by an amino acid antiporter. Nature 463: 828–832. Available: http://dx.doi.org/10.1038/nature08741. Accessed 30 October 2013.

4. Ma D, Lu P, Yan C, Fan C, Yin P, et al. (2012) Structure and mechanism of a glutamate–GABA antiporter. Nature 483: 632–636. Available: http://dx.doi.org/10.1038/nature10917.

5. Lomize MA, Lomize AL, Pogozheva ID, Mosberg HI (2006) OPM: Orientations of Proteins in Membranes database. Bioinformatics 22: 623–625. Available: http://www.ncbi.nlm.nih.gov/pubmed/16397007. Accessed 12 September 2014.

6. Jo S, Kim T, Iyer VG, Im W (2008) CHARMM-GUI: A web-based graphical user interface for CHARMM. J Comput Chem 29: 1859–1865. Available: http://www.ncbi.nlm.nih.gov/pubmed/18351591. Accessed 22 November 2013.

7. Humphrey W, Dalke A, Schulten K (1996) VMD: Visual molecular dynamics. J Mol Graph 14: 33–38. Available: http://www.ncbi.nlm.nih.gov/pubmed/8744570.

8. Ullmann RT, Ullmann GM (2012) GMCT : A Monte Carlo simulation package for macromolecular receptors. J Comput Chem 33: 887–900. Available: http://www.ncbi.nlm.nih.gov/pubmed/22278916. Accessed 30 October 2013.

9. Gao X, Lu F, Zhou L, Dang S, Sun L, et al. (2009) Structure and mechanism of an amino acid antiporter. Science 324: 1565–1568. Available: http://www.ncbi.nlm.nih.gov/pubmed/19478139. Accessed 30 October 2013.

10. Fang Y, Jayaram H, Shane T, Kolmakova-Partensky L, Wu F, et al. (2009) Structure of a prokaryotic virtual proton pump at 3.2 Å resolution. Nature 460: 1040–1043. Available: http://www.pubmedcentral.nih.gov/articlerender.fcgi?artid=2745212&tool=pmcentrez&rendertype=abstract. Accessed 30 October 2013.

11. Fang Y, Kolmakova-Partensky L, Miller C (2007) A Bacterial Arginine-Agmatine Exchange Transporter Involved in Extreme Acid Resistance. J Biol Chem 282: 176–182. Available: http://www.ncbi.nlm.nih.gov/pubmed/17099215. Accessed 30 October 2013.

12. Phillips JC, Braun R, Wang W, Gumbart J, Tajkhorshid E, et al. (2005) Scalable molecular dynamics with NAMD. J Comput Chem 26: 1781–1802. Available: http://www.pubmedcentral.nih.gov/articlerender.fcgi?artid=2486339&tool=pmcentrez&rendertype=abstract. Accessed 30 October 2013.

13. Feller SE, MacKerell AD (2000) An Improved Empirical Potential Energy Function for Molecular Simulations of Phospholipids. J Phys Chem B 104: 7510–7515. Available: http://dx.doi.org/10.1021/jp0007843. Accessed 22 July 2014.

14. MacKerell AD, Bashford D, Bellott M, Dunbrack RL, Evanseck JD, et al. (1998) All-atom empirical potential for molecular modeling and dynamics studies of proteins. J Phys Chem B 102: 3586–3616. Available: http://dx.doi.org/10.1021/jp973084f. Accessed 17 July 2014.

15. MacKerell AD, Feig M, Brooks CL (2004) Extending the treatment of backbone energetics in protein force fields: limitations of gas-phase quantum mechanics in reproducing protein conformational distributions in molecular dynamics simulations. J Comput Chem 25: 1400–1415. Available: http://www.ncbi.nlm.nih.gov/pubmed/15185334. Accessed 15 July 2014.

16. Hénin J, Shinoda W, Klein ML (2008) United-atom acyl chains for CHARMM phospholipids. J Phys Chem B 112: 7008–7015. Available: http://www.ncbi.nlm.nih.gov/pubmed/18481889. Accessed 22 July 2014.

17. Darden T, York D, Pedersen L (1993) Particle mesh Ewald: An N⋅log(N) method for Ewald sums in large systems. J Chem Phys 98: 10089. Available: http://scitation.aip.org/content/aip/journal/jcp/98/12/10.1063/1.464397.

18. Tuckerman M, Berne BJ, Martyna GJ (1992) Reversible multiple time scale molecular dynamics. J Chem Phys 97: 1990. Available: http://scitation.aip.org/content/aip/journal/jcp/97/3/10.1063/1.463137. Accessed 11 July 2014.

19. Andersen HC (1983) Rattle: A “velocity” version of the shake algorithm for molecular dynamics calculations. J Comput Phys 52: 24–34. Available: http://adsabs.harvard.edu/abs/1983JCoPh..52...24A. Accessed 24 August 2014.
