# Supplementary figures and images for: Unveiling the Mechanism of Arginine Transport through AdiC with Molecular Dynamics Simulations: The Guiding Role of Aromatic Residues
Source: PLoS One. 2016 Aug 2;11(8):e0160219. doi: 10.1371/journal.pone.0160219 (PMC4970712; doi:10.1371/journal.pone.0160219)

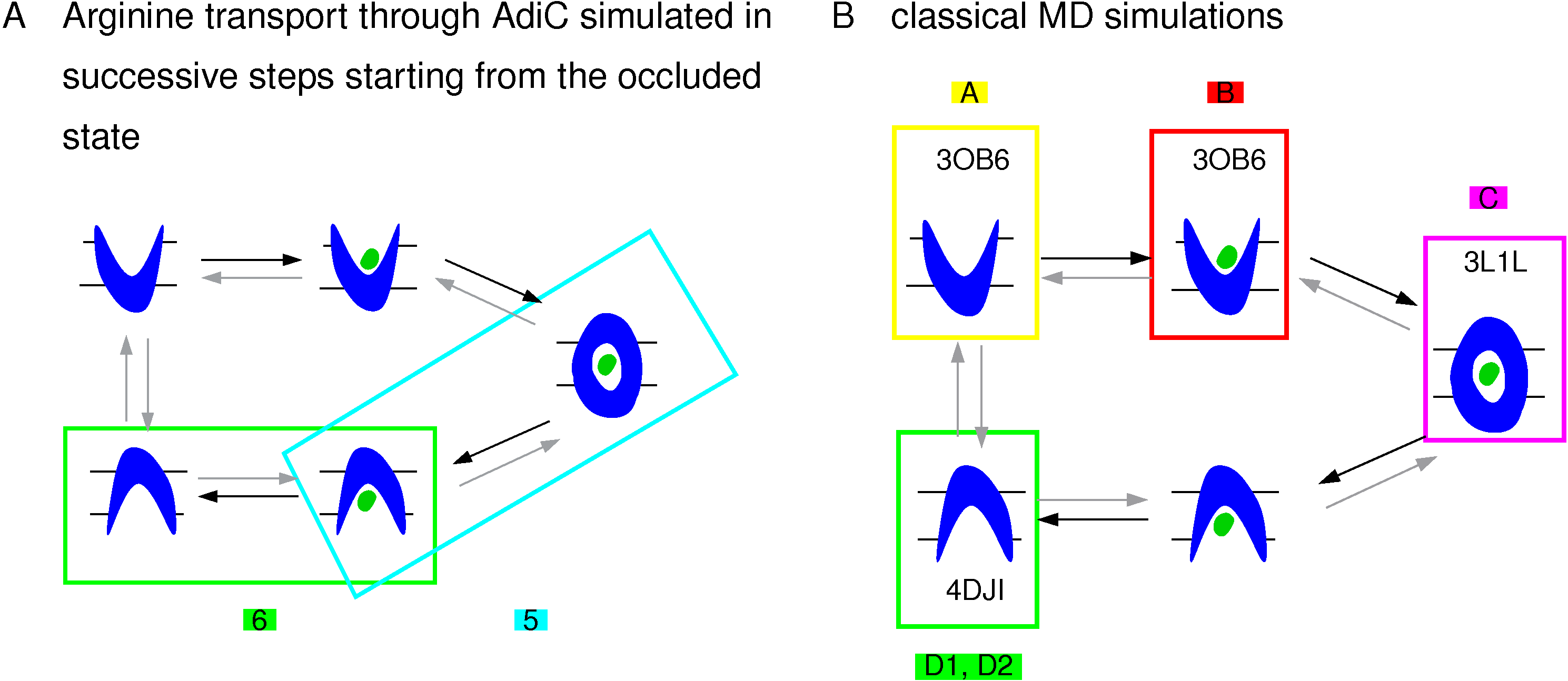

Supplement: S1 Fig — (A) Reconstruction of truncated arginine transport by AdiC starting from the AdiC occluded substrate-bound structure using tMDs followed by relaxation MD simulations: (Step 5) Transition from the occluded substrate-bound to the IF open substrate-bound state targeting each of the two IF open crystal structures of GadC (PDB IDs: 4DJI or 4DJK [27]) including Trp293 side chain in the targeted protein portions (for further details see Material and Methods and S2 Table). (Step 6) Release of the arginine substrate starting from the final conformation of the tMDs obtained from step 5. Black arrows indicate the migration direction of the arginine substrate. (B) Conventional MD simulations performed on AdiC and GadC crystal structures adopting different conformational states: the AdiC OF open arginine-bound structure without and with the arginine substrate (simulation A and B, respectively), the occluded arginine-bound structure (simulation C) and the GadC IF open substrate-free structure with (simulation D1) and without its C-plug (simulation D2) (for further details see Material and Methods and S3 Table). (TIFF) [file pone.0160219.s003.tiff]

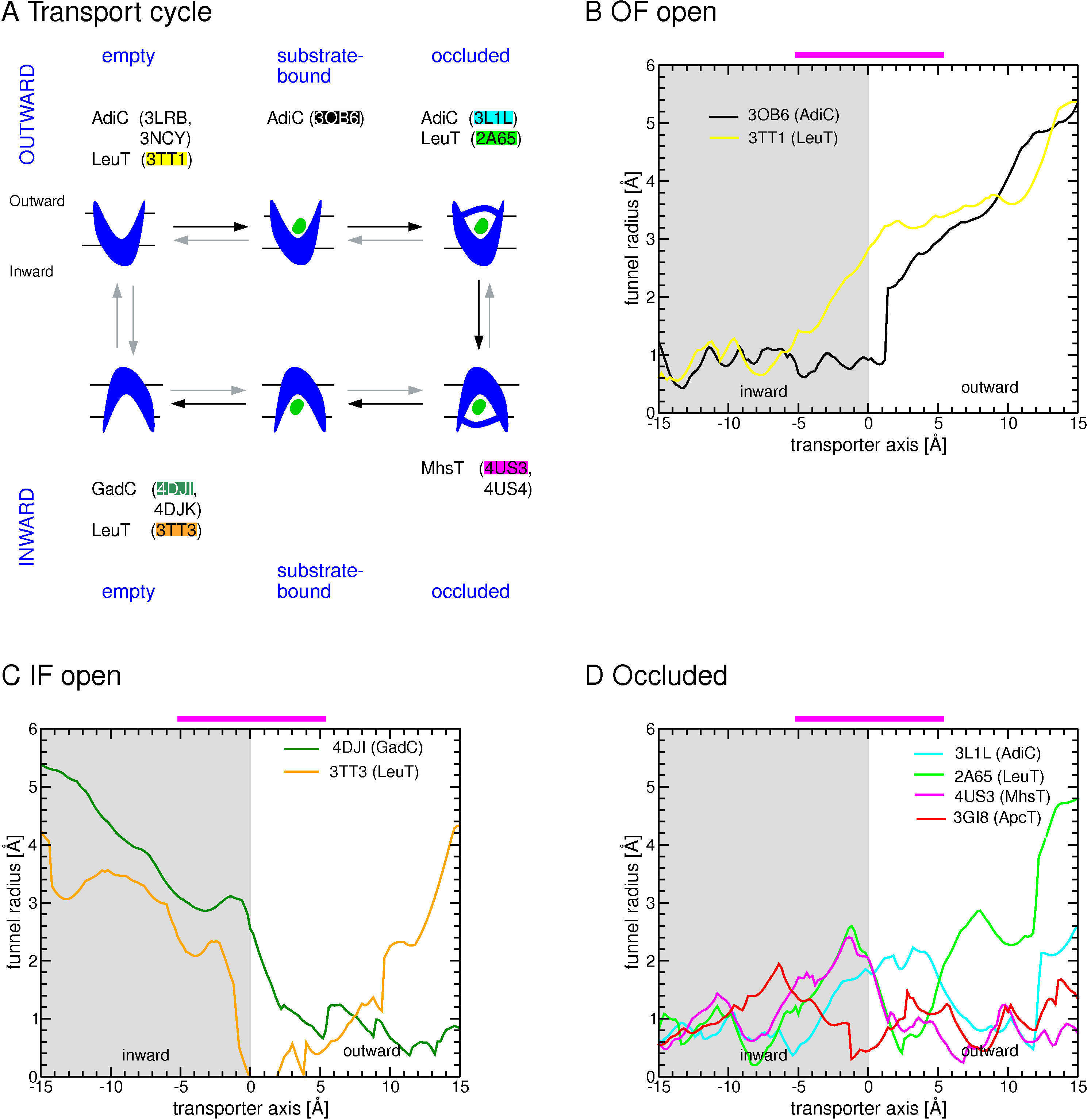

Supplement: S2 Fig — (A) A schematic representation of the transport cycle featuring a series of potential conformational states. Substrate (green ellipsoid) binding to the OF open state promotes occlusion of the transporter. A potential OF occluded state undergoes a subsequent conformational change that switches it into an IF occluded state. Opening of the binding site towards the IF side leads to the release of the substrate. Other intermediate states may be sampled along the transport cycle such as the IF occluded substrate free state that was proposed to be adopted by ApcT (PDB ID: 3GI8 [33]). In antiporters, the return from the IF open to the OF open unbound structure requires the binding and transport of a second substrate but in the opposite direction. Each of the potential conformational states is exemplified by crystal structures (identified with their PDB IDs) either of the AdiC and GadC AR antiporters [14,15,25–27], LeuT [16,30] or MhsT [23] and taken from published reports. Black arrows indicate the migration direction of the arginine substrate. (B-D) The profile of the funnel radius, computed for crystal structures of amino acid transporters belonging to the APC superfamily selected from panel A, as a function of position along their main axis. Panel D combines both the OF and IF occluded states and also includes the funnel radius profile computed for ApcT used as an example of an IF occluded substrate-free structure. The binding site region (-5 to 5 Å) is highlighted by a magenta bar in (B-D). (TIFF) [file pone.0160219.s004.tiff]

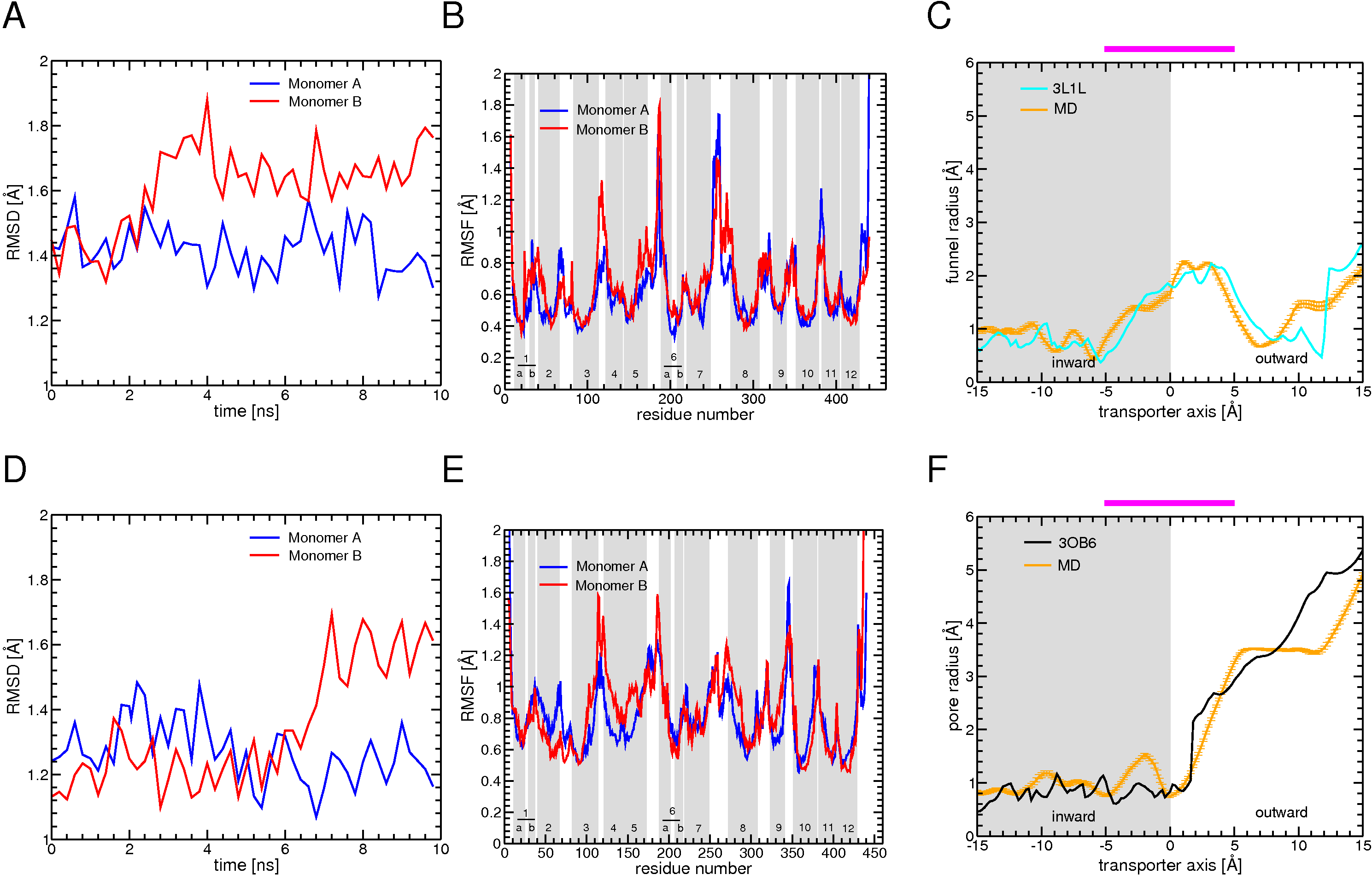

Supplement: S3 Fig — The latter was performed after removal of the arginine ligand. (A) Time evolution of the backbone RMSD of the simulations of the occluded structure (S1B Fig: simulation C). (B) Backbone positional RSMF of the monomer A (blue) and monomer B (red) of the trajectory of the occluded structure. (B) The profile of the funnel radius as a function of the position along the main axis of the transporter, averaged over the last 0.1 ns of the 10-ns conventional simulation of the occluded structure (orange). For comparison the profile is also depicted for the occluded crystal structure (3L1L, cyan). (D) Time evolution of the backbone RMSD of the simulations of the OF open state (S1B Fig: simulation A). (E) Backbone positional RSMF of the monomer A (blue) and monomer B (red) of the trajectory of the OF open structure. (F) The profile of the funnel radius as a function of position along the main axis of the transporter, averaged over the last 0.1 ns of the conventional simulation of the OF open structure (orange). For comparison the profile is also depicted for the OF open crystal structure (3OB6, black). In B and D transmembrane helical regions are highlighted by grey stripes. In E and F the standard errors are shown as orange bars. The simulation of the OF open substrate-bound structure was performed after removal of the arginine ligand. The binding site region (-5 to 5 Å) is highlighted by a magenta bar in (C) and (F). (TIFF) [file pone.0160219.s005.tiff]

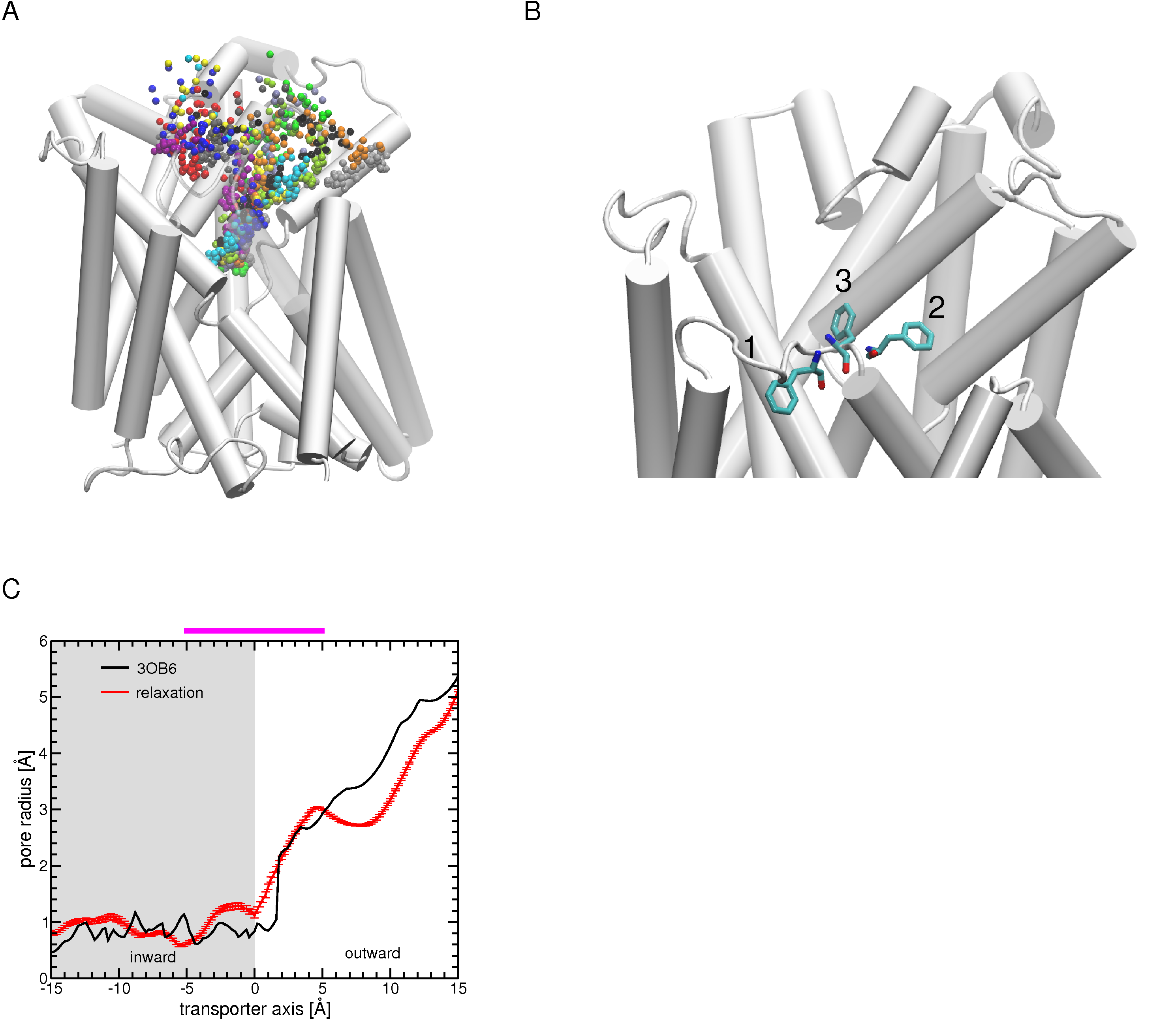

Supplement: S4 Fig — (A) Arginine binding pathways observed in the twelve different monomers from the six tMDs are depicted by spheres marking the positions of the Cβ atom of Arg+ using snapshots extracted every 50 ps. The protein is shown as a white cartoon. Each ligand trajectory is colored differently. (B) Reorientation of Phe350 during the MD trajectory as shown by its starting position (1) in the AdiC OF open substrate-bound crystal structure (PDB ID: 3OB6 [15]) and at the end of the MD simulation (3). The location of Phe350 in the OF open substrate-free crystal structure (2) (PDB ID: 3LRB [25]) is also shown as a reference. Phe350 is depicted as sticks and the protein as white cartoon. (C) The profile of the funnel radius as a function of the position along the main axis of the transporter for one of the twelve monomers in the six tMDs of the dimers averaged over the last 0.1 ns of the relaxation trajectory (red). For comparison the profile is also depicted for the OF open crystal structure (3OB6, black). The standard errors are shown as red bars. The binding site region (-5 to 5 Å) is highlighted by a magenta bar. (TIFF) [file pone.0160219.s006.tiff]

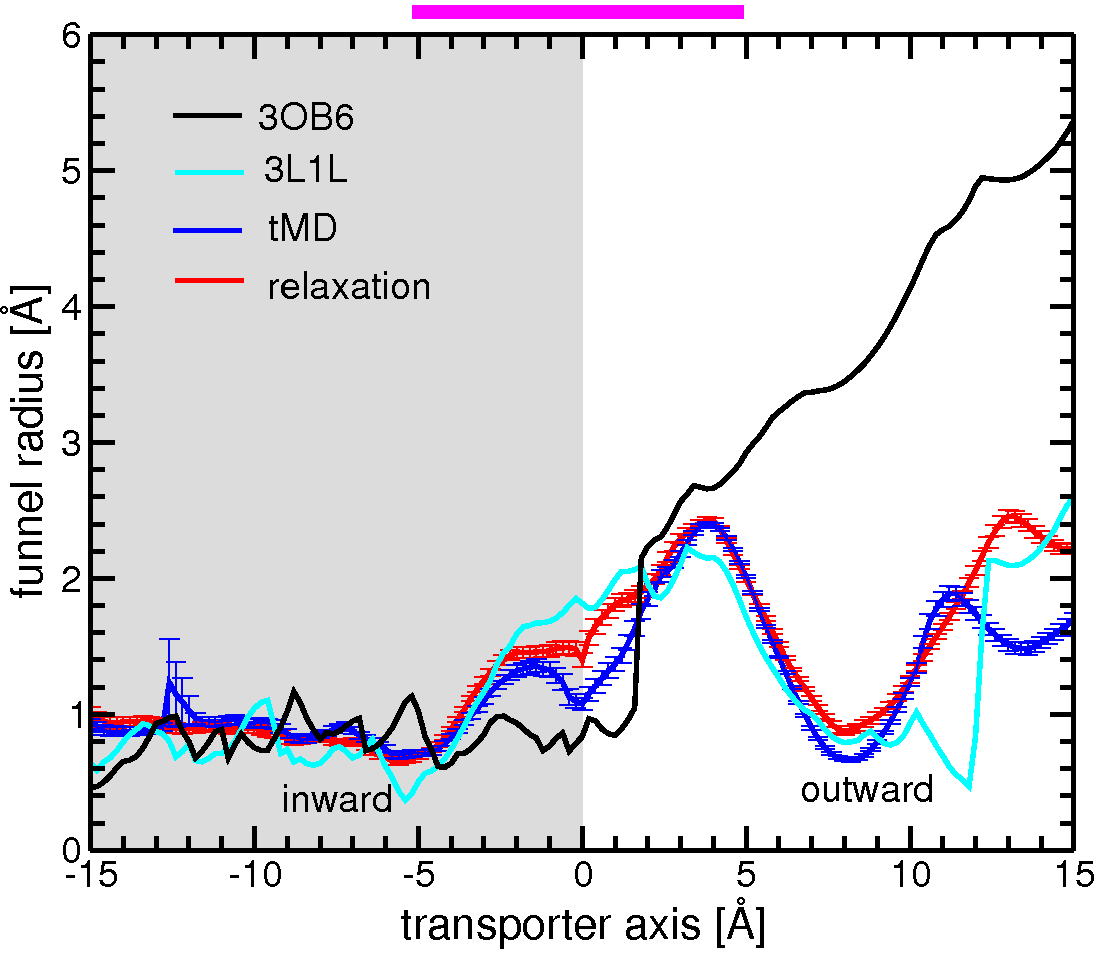

Supplement: S5 Fig — The profile of the funnel radius as a function of the position of the main axis of the transporter, averaged over the last 0.1 ns of one tMD (blue) and of its subsequent relaxation (red) simulations including Trp202 in the targeted ensemble of atoms (see main text). The standard errors are shown as bars. The radius profile is also depicted for the OF open (3OB6, black) and occluded (3L1L, cyan) substrate-bound crystal structures. The binding site region (-5 to 5 Å) is highlighted by a magenta bar. (TIFF) [file pone.0160219.s007.tiff]

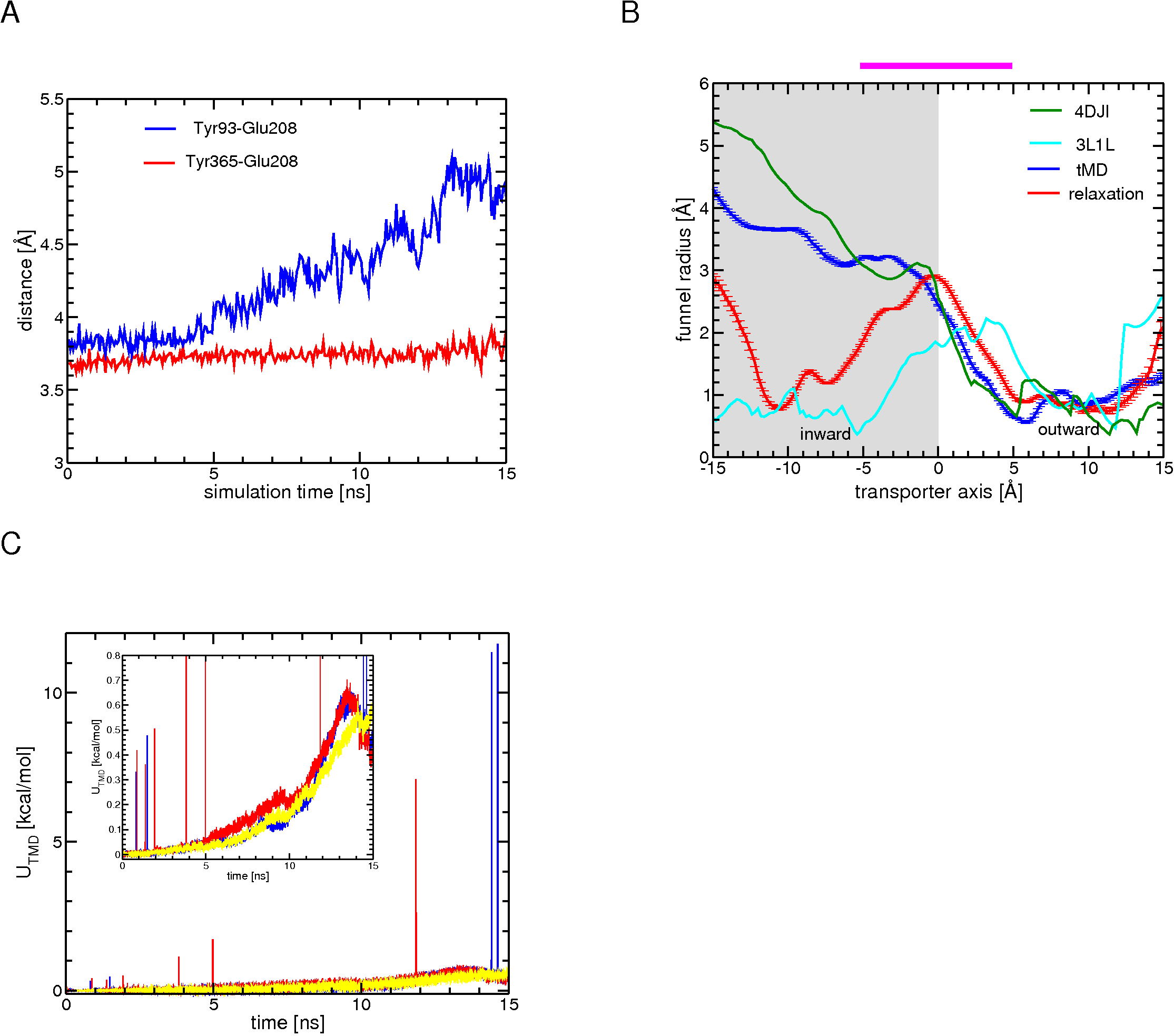

Supplement: S6 Fig — (A) Evolution of the distance between the Cδ atom of Glu208 and either the Tyr93 (blue) or Tyr365 (red) side chain hydroxyl oxygen atom averaged over the tMD trajectories. (B) The profile of the funnel radius as a function of the position along the main axis of the transporter, averaged over the last 0.1 ns of one tMD (blue) and of its subsequent relaxation MD (red) simulations. The standard errors are shown as bars. The profile is also depicted for the occluded AdiC (3L1L, cyan) and the IF open (4DIJ, green) GadC crystal structure. The binding site region (-5 to 5 Å) is highlighted by a magenta bar. (C) The restraint potential UTMD is shown for three different trajectories (blue, red, and yellow curves) as a function of the tMD simulation time. An inlay shows a close-up of the curve between 0 and 0.6 kcal/mol. Vertical peaks occur when the instantaneous best-fit RMSD of the current coordinates are quite different from the target coordinates leading to a high restrained potential. The different curves correspond to a simulation in which, during the relaxation MD following the TMD, i) the two monomers close (yellow), ii) one monomer closes and the other remains open (blue) and iii) both monomers remain open (red). (TIFF) [file pone.0160219.s008.tiff]

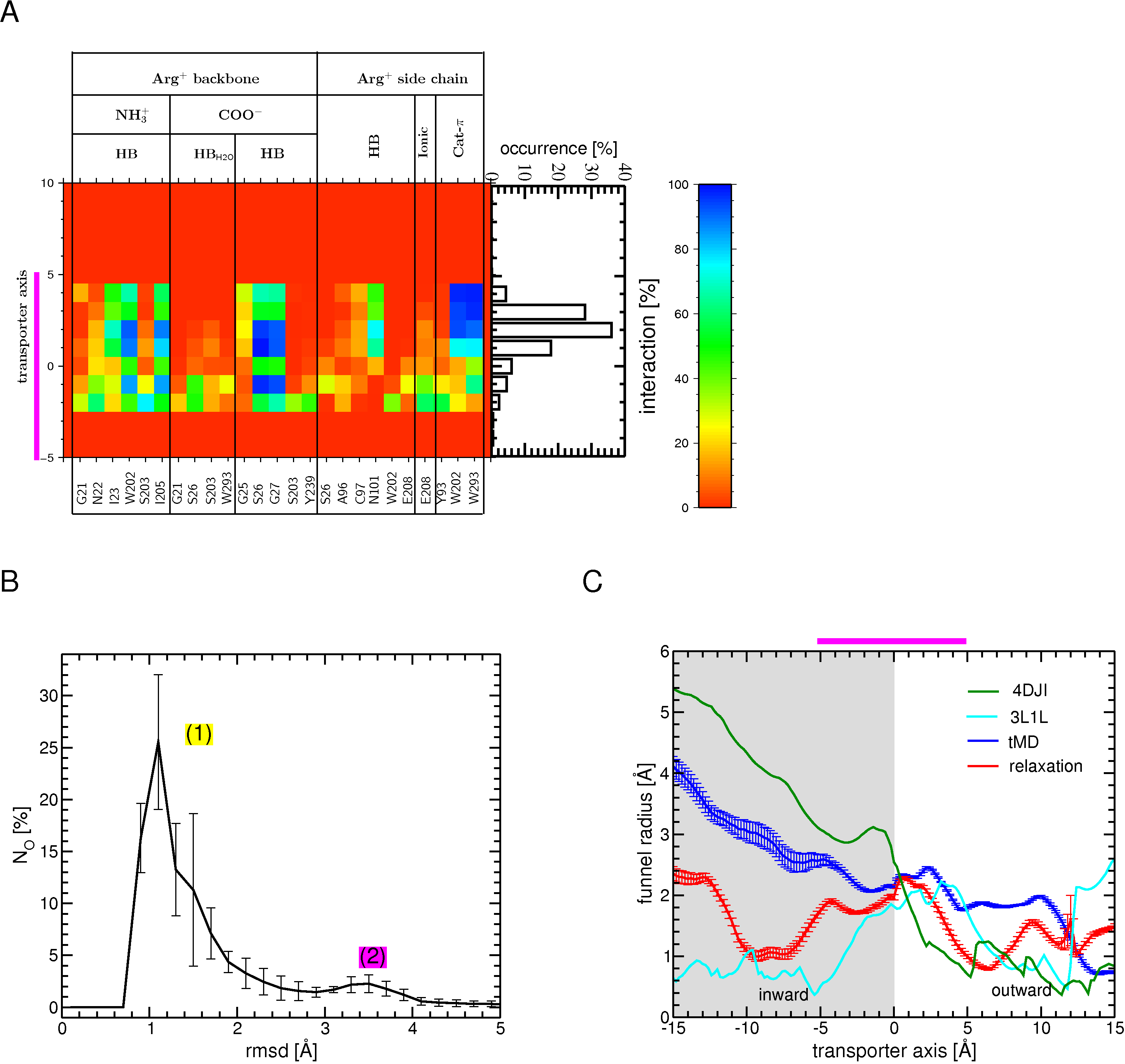

Supplement: S7 Fig — (A) Interactions (H bonds, ionic and cation-π) formed between the Arg+ backbone and side chain and protein residues during the tMDs. Only interactions with an occurrence higher than 20% in at least one bin width from all 12 binding events of the 6 tMD trajectories are shown. The abbreviations used for the different interactions are listed in the legend of Fig 3. The binding site region (-5 to 5 Å) is highlighted by a magenta bar. (B) Number of occurrences (NO) for finding the substrate at a certain RMSD value computed for the carbon atoms of Arg+ using all tMD simulations and its crystal position in 3L1L as a reference. The standard errors are shown as bars. The RMSD values corresponding to the two representative positions of Arg+ shown in Fig 6C are numbered accordingly. (C) The profile of the funnel radius as a function of the position along the main axis of the transporter, averaged over the last 0.1 ns of one tMD (blue) and of its following relaxation simulation (red). The standard errors are shown as bars. The profile is also depicted for the IF open GadC (4DJI, green) and the OF occluded AdiC crystal structure (3L1L, cyan). (TIFF) [file pone.0160219.s009.tiff]

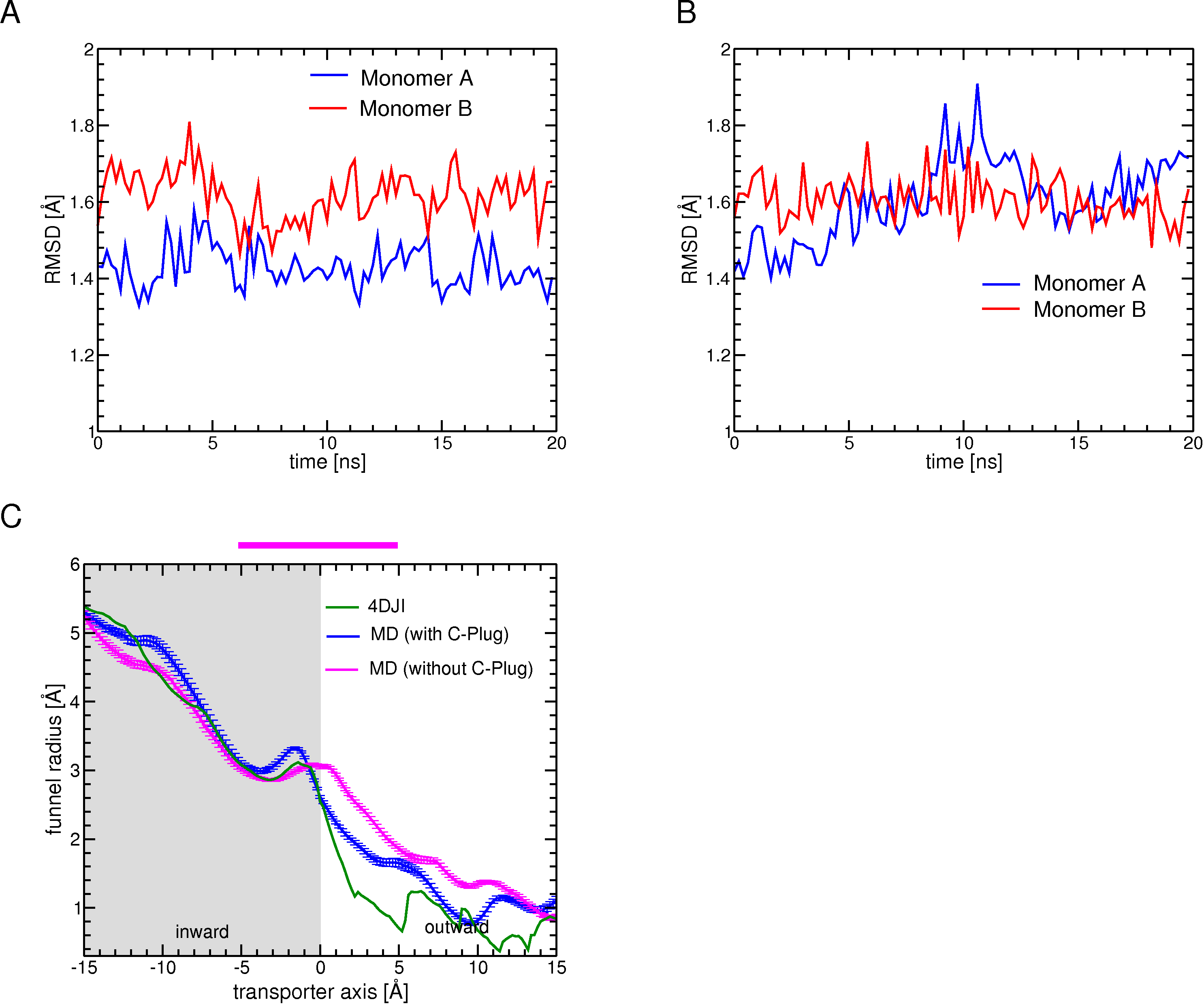

Supplement: S8 Fig — (A-B) Time evolution of the backbone RMSD of the 10-ns long GadC simulations (A) with and (B) without the plug. (C) The profile of the funnel radius as a function of the position along the main axis of the transporter (PDB ID: 4DJI [27]), averaged over the last 0.1 ns of the MD simulations with (blue) or without (magenta) the C-plug. The standard errors are shown as bars. The profile is also depicted for the GadC IF crystal structure (4DJI, green). The binding site region (-5 to 5 Å) is highlighted by a magenta bar. (TIFF) [file pone.0160219.s010.tiff]

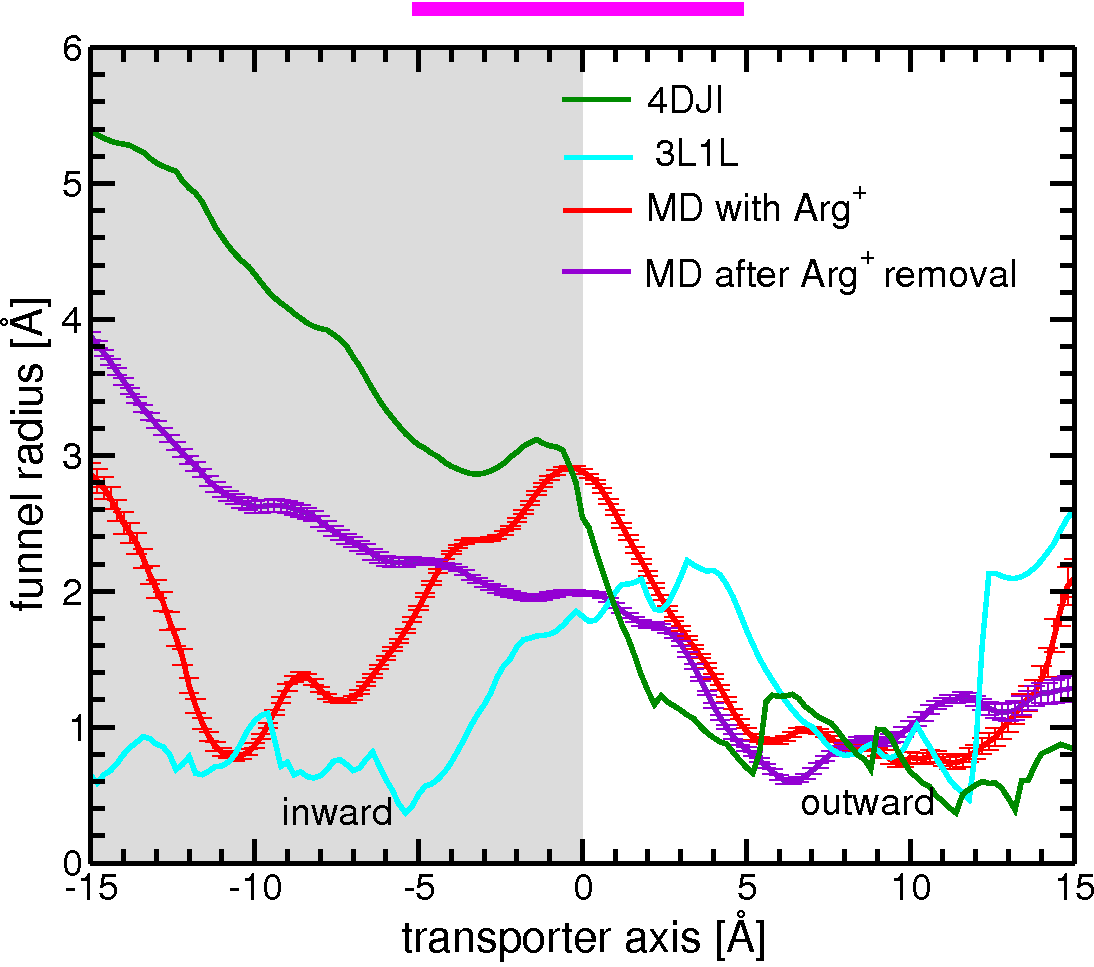

Supplement: S9 Fig — The profile of the funnel radius as a function of the position of the main axis of the transporter, averaged over the last 0.1 ns of one tMD simulation (violet). The standard errors are shown as bars. For the sake of comparison, the profile is also depicted averaged over the last 0.1 ns of one of the relaxation trajectories (red) simulating the transition from the occluded to IF open Arg+-bound states (Fig 2A: step3b) as well as for the IF open GadC (4DJI, green) and the OF occluded AdiC (3L1L, cyan) crystal structure. The binding site region (-5 to 5 Å) is highlighted by a magenta bar. (TIFF) [file pone.0160219.s011.tiff]

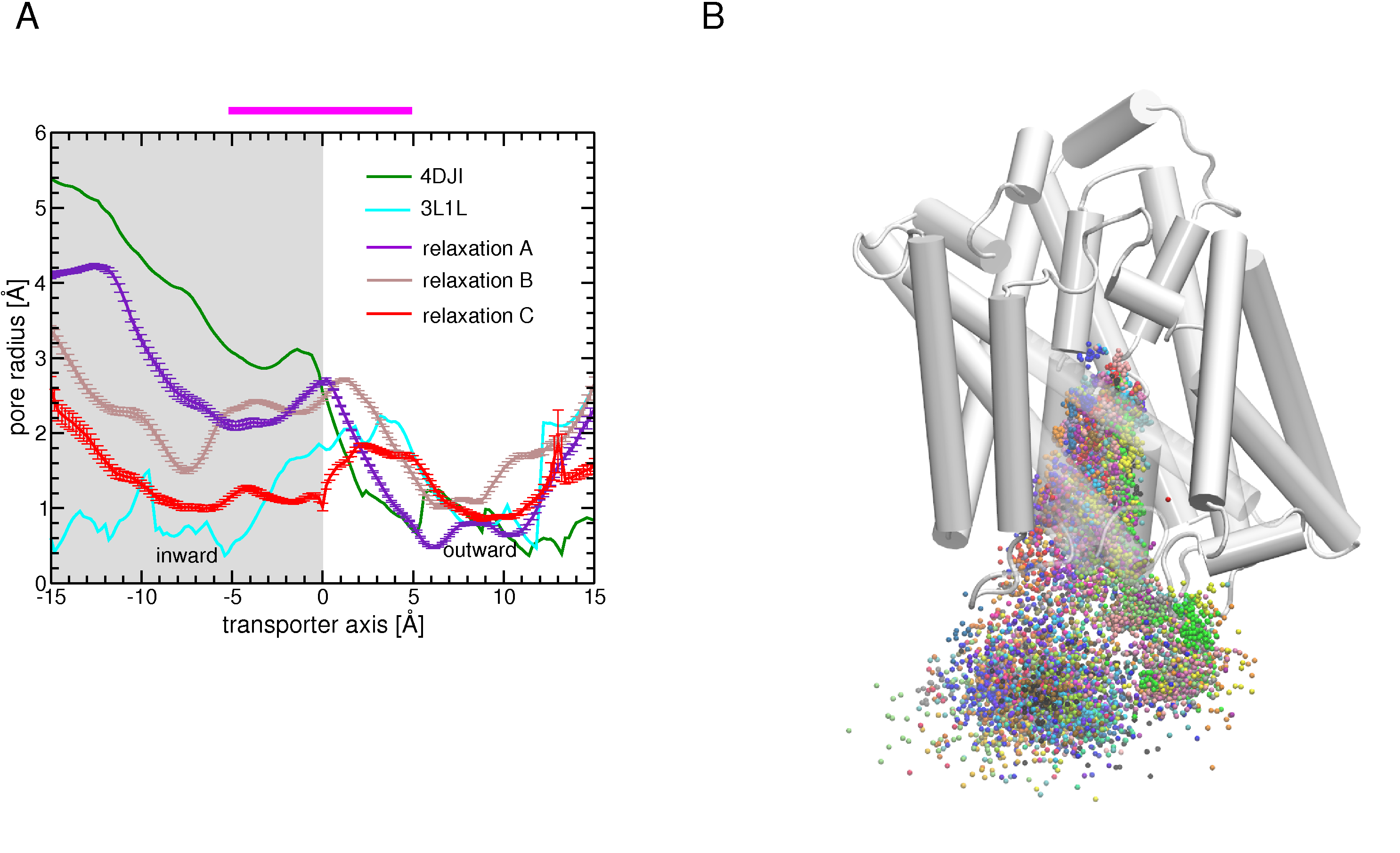

Supplement: S10 Fig — (A) The profile of the funnel radius as a function of the position along the main axis of the transporter, averaged over the last 0.1 ns of different relaxation simulations features either an IF quasi-open (violet), semi-open (brown) or closed (red) state. The standard errors are shown as bars. For the sake of comparison, the profile is also depicted for the IF open GadC (4DJI, green) and the OF occluded AdiC (3L1L, cyan) crystal structure. The binding site region (-5 to 5 Å) is highlighted by a magenta bar. (B) Arginine release pathways in the 72 different monomers during the 36 tMDs are depicted by spheres marking the positions of the Cβ atom of the ligand using snapshots extracted every 50 ps from the tMD trajectories. The protein is shown as a white cartoon. Each ligand trajectory is colored differently. (TIFF) [file pone.0160219.s012.tiff]
