# Supplementary material for: Unveiling the Mechanism of Arginine Transport through AdiC with Molecular Dynamics Simulations: The Guiding Role of Aromatic Residues
Source: PLoS One. 2016 Aug 2;11(8):e0160219. doi: 10.1371/journal.pone.0160219 (PMC4970712; doi:10.1371/journal.pone.0160219)
Supplement: S1 Table — Simulated steps are numbered according to Fig 2 (see main text). The simulations started with the binding of Arg+ to the OF open structure using two different initial positions (Conf1, Conf2) of the protein extracted from a classical MD simulation of the OF open crystal structure after removal of the ligand (S1B Fig: simulation B) and three different starting positions for Arg in the external medium leading to a total of 6 simulations for this step. Either classical MD (step 1a) or tMD followed by relaxation MD simulations (step 1b) were performed. The occlusion was modeled targeting the occluded substrate-bound AdiC crystal structure including (step 2b) or not (step 2a) W202 in the targeted ensemble of atoms. The transition to the IF open state was first simulated using the GadC crystal structure 4DJI as a guide (step 3a) (see Material and Methods for detail). A second series of simulations exploited protein portions from either of the two GadC crystal structures (4DJI or 4DJK) and included in both simulations W293 in the targeted ensemble of atoms (see Material and Methods) leading to a total of 24 simulations. The release of Arg+ to the cytosol was simulated starting from the last conformation of the tMDs produced from step 3b and three different positions of the arginine located in the cytosol were targeted leading to an ensemble of 72 simulations. (DOCX) [file pone.0160219.s013.docx]

| Step | | 1a | 1b | 2a | 2b | 3a | 3b | 4 | |
| --- | --- | --- | --- | --- | --- | --- | --- | --- | --- |
| Process | | Arg^+^ binding to OF open | | Occlusion | | Transition from occluded to IF open | | Arg^+^ release to the IF side | |
|  |  |  |  | -W202 | +W202 | -W293 | +W293 |  |  |
| Simulation type | | MD | tMD + relaxation | tMD + relaxation | | tMD + relaxation | | tMD + relaxation | |
| Time [ns] | | 10 | 5 +5 | 10 + 5 | | 15 + 5 | | 10 + 5 | |
| Conf1 | 3 Ligand positions |  |  |  |  |  |  | 3 Ligand positions |  |
|  |  |  |  |  |  |  |  |  |  |
|  |  |  |  |  |  |  |  |  |  |
| Conf2 | 3 Ligand positions |  |  |  |  |  |  |  |  |
|  |  |  |  |  |  |  |  |  |  |
|  |  |  |  |  |  |  |  |  |  |
| Total number of simulations | | 6 | 6 | 3 | 6 | 3 | 12 | 36 | |
