# Supplementary material for: Unveiling the Mechanism of Arginine Transport through AdiC with Molecular Dynamics Simulations: The Guiding Role of Aromatic Residues
Source: PLoS One. 2016 Aug 2;11(8):e0160219. doi: 10.1371/journal.pone.0160219 (PMC4970712; doi:10.1371/journal.pone.0160219)
Supplement: S2 Table — The simulations are summarized in S1A Fig. Three different conformations of the AdiC-Arg+ complex (Conf1, Conf2, Conf3) were extracted from a classical MD simulation of the occluded substrate-bound AdiC crystal structure (S1B Fig: simulation C; S3 Table). For the transition from the occluded to the IF open state (step 5) protein portions from either of the two GadC crystal structures (4DJI or 4DJK) were targeted (for more details see Material and Methods) leading to 6 different simulations. For the simulations of the Arg+ release to the IF side (step 6) three different positions of the arginine located in the cytosol were targeted leading to a total of 18 simulations for this step. (DOCX) [file pone.0160219.s014.docx]

| Step | 5 | 6 |
| --- | --- | --- |
| Process | Transition from occluded to IF open | Arg^+^ release to the IF side |
| Simulation type | tMD + relaxation | tMD + relaxation |
| Time [ns] | 15 + 5 | 10 + 5 |
| Conf1 |  |  |
| Conf2 |  |  |
| Conf3 |  |  |
| Total number of simulations | 6 | 18 |
