# Supplementary material for: Unveiling the Mechanism of Arginine Transport through AdiC with Molecular Dynamics Simulations: The Guiding Role of Aromatic Residues
Source: PLoS One. 2016 Aug 2;11(8):e0160219. doi: 10.1371/journal.pone.0160219 (PMC4970712; doi:10.1371/journal.pone.0160219)
Supplement: S3 Table — (DOCX) [file pone.0160219.s015.docx]

| Simulation | A | B | C | D1 | D2 |
| --- | --- | --- | --- | --- | --- |
|  | OF open substrate-free | OF open substrate-bound | Occluded substrate-bound | IF open substrate-free | IF open substrate-free without C-plug |
| Protein | AdiC | AdiC | AdiC | GadC | GadC |
| PDB ID | 3OB6 | 3OB6 | 3L1L | 4DJI | 4DJI |
| Time [ns] | 10 ns | 10 ns | 10 ns | 20 ns | 20 ns |
| Total number of simulation | 1 | 1 | 1 | 1 | 1 |
