# Supplementary material for: Unveiling the Mechanism of Arginine Transport through AdiC with Molecular Dynamics Simulations: The Guiding Role of Aromatic Residues
Source: PLoS One. 2016 Aug 2;11(8):e0160219. doi: 10.1371/journal.pone.0160219 (PMC4970712; doi:10.1371/journal.pone.0160219)
Supplement: S4 Table — The simulations were performed after removal of arginine and filling the binding site with water molecules (Fig 2A: step 3b). (DOCX) [file pone.0160219.s016.docx]

| Simulation starting from the end conformation of | | the tMD targeting 4DIJ | the tMD targeting 4DIK |
| --- | --- | --- | --- |
| Time [ns] | | MD  10 | MD  10 |
| Conf1 | 3 Ligand positions |  |  |
|  |  |  |  |
|  |  |  |  |
| Conf2 | 3 Ligand positions |  |  |
|  |  |  |  |
|  |  |  |  |
| Total number of simulations | | 6 | 6 |
