# Supplementary material for: Unveiling the Mechanism of Arginine Transport through AdiC with Molecular Dynamics Simulations: The Guiding Role of Aromatic Residues
Source: PLoS One. 2016 Aug 2;11(8):e0160219. doi: 10.1371/journal.pone.0160219 (PMC4970712; doi:10.1371/journal.pone.0160219)
Supplement: S5 Table — The electrostatic interactions have been calculated using vmd [57]. All other interactions were identified using eucb [59]. The abbreviations used for the different interactions in this table and different figures are: ionic for ionic interaction, HB for hydrogen bond, HBH2O for water-mediated hydrogen bond, and Cat-π for cation π interaction. (DOCX) [file pone.0160219.s017.docx]

| Interaction with | Type | Definition of the interaction |
| --- | --- | --- |
| Substrate Backbone  COO^-^ group | Ionic | d ≤ 6 Å between any side chain nitrogen atoms of protein Arg nd Lys residues and at least one of the substrate arginine backbone oxygen atoms. |
|  | HB | d ≤ 3.5 Å between any H bond donor oxygen, nitrogen or sulfur atom of protein residues and at least one of the substrate arginine backbone oxygen atoms and an angle α (H bond donor - Hydrogen - H bond acceptor) > 120°. |
|  | HB_H2O_ | d ≤ 3.5 Å between a water oxygen atom and at least one of the substrate arginine backbone oxygen atoms and an angle α (H bond donor - Hydrogen - H bond acceptor) > 120°. This water oxygen atom must simultaneously be at a distance d ≤ 3.5 Å of any oxygen, nitrogen or sulfur H bond acceptor of protein residues and an angle α > 120°. |
| Substrate backbone NH_3_^+^group | Ionic | d ≤ 6 Å between any side chain oxygen atom of protein Asp and Glu residues and one of the substrate arginine backbone nitrogen atom. |
|  | HB | d ≤ 3.5 Å between any H bond acceptor oxygen, nitrogen or sulfur atom of protein residues and the arginine backbone nitrogen atom and an angle α (H-bond donor - Hydrogen - H-bond acceptor) α > 120°. |
|  | HB_H2O_ | d ≤ 3.5 Å between a water oxygen atom and the arginine backbone nitrogen atom and an angle α (H-bond donor - Hydrogen - H-bond acceptor) > 120°. This water oxygen atom must simultaneously be at a distance d ≤ 3.5 Å of any oxygen, nitrogen or sulfur H bond acceptors of protein residues and an angle α > 120°. |
|  | Cat-π | d ≤ 6 Å between the center of mass of three selected heavy atoms of the aromatic rings of Trp, Phe and Tyr and the arginine backbone nitrogen atom and an angle β between the aromatic plane and the plane spanned by at least one arginine nitrogen and one of its corresponding hydrogen atom is higher than 60°. |
| Substrate side chain | Ionic | d ≤ 6 Å between at least one side chain oxygen atom of protein Asp and Glu residues and the arginine side chain nitrogen atoms. |
|  | HB | d ≤ 3.5 Å between any oxygen, nitrogen or sulfur H bond acceptor of the protein residues and the arginine side chain nitrogen atoms and an angle α (H bond donor - Hydrogen – H bond acceptor) α > 120°. |
|  | HB_H2O_ | d ≤ 3.5 Å between a water oxygen atom and the arginine side chain nitrogen atom and an angle α (H-bond donor - Hydrogen - H-bond acceptor) > 120°. At the same time this water atom must simultaneously be at a distance d ≤ 3.5 Å of any oxygen, nitrogen or sulfur H bond acceptor of protein residues and α > 120°. |
|  | Cat-π | d ≤ 6 Å between the center of mass of three selected heavy atoms of the aromatic rings of Trp, Phe and Tyr and any arginine side chain nitrogen atom and an angle β between the aromatic plane and the plane spanned by at least one of the arginine side chain nitrogen atoms and one of its corresponding hydrogen atom is either > 60 or < 30°. |
