# Supplementary material for: Unveiling the Mechanism of Arginine Transport through AdiC with Molecular Dynamics Simulations: The Guiding Role of Aromatic Residues
Source: PLoS One. 2016 Aug 2;11(8):e0160219. doi: 10.1371/journal.pone.0160219 (PMC4970712; doi:10.1371/journal.pone.0160219)
Supplement: S6 Table — (DOCX) [file pone.0160219.s018.docx]

| AdiC | PotE (32%) | CadB (38%) | GadC (21%) | Yeast Arg transporter (Can1) (20%) |
| --- | --- | --- | --- | --- |
| Phe350 | Phe351 | Phe349 | Phe367 | Phe449 |
| Trp202 | Trp198 | Trp201 | Leu212 | Phe295 |
| Trp293 | Trp289 | Trp292 | Trp308 | Trp177* |
| Tyr93 | Tyr89 | Tyr92 | Tyr96^§^ | Tyr173 |

*Conservation of AdiC Trp293 in Can1 (Trp177) is visible only when the 3D structures of Can1 and AdiC are superimposed.

^§^In the sequence alignment Tyr96 (GadC) is shifted by one residue relative to Tyr93 (AdiC). The examination of the structures points to the possibility of the Tyr96 (GadC) sidechain to make interactions similar to Tyr93 (AdiC) sidechain.
